# Composition and functional diversity of bacterial communities during swine carcass decomposition

**DOI:** 10.5713/ab.23.0140

**Published:** 2023-06-26

**Authors:** Michelle Miguel, Seon-Ho Kim, Sang-Suk Lee, Yong-Il Cho

**Affiliations:** 1 Department of Animal Science and Technology, Sunchon National University, Suncheon, Jeonnam 57922, Korea

**Keywords:** Bacterial Community, Decomposition, Microcosm, Soil Burial, Swine Carcass

## Abstract

**Objective:**

This study investigated the changes in bacterial communities within decomposing swine microcosms, comparing soil with or without intact microbial communities, and under aerobic and anaerobic conditions.

**Methods:**

The experimental microcosms consisted of four conditions: UA, unsterilized soil–aerobic condition; SA, sterilized soil–aerobic condition; UAn, unsterilized soil–anaerobic condition; and San, sterilized soil–anaerobic condition. The microcosms were prepared by mixing 112.5 g of soil and 37.5 g of ground carcass, which were then placed in sterile containers. The carcass-soil mixture was sampled at day 0, 5, 10, 30, and 60 of decomposition, and the bacterial communities that formed during carcass decomposition were assessed using Illumina MiSeq sequencing of the 16S rRNA gene.

**Results:**

A total of 1,687 amplicon sequence variants representing 22 phyla and 805 genera were identified in the microcosms. The Chao1 and Shannon diversity indices varied in between microcosms at each period (p<0.05). Metagenomic analysis showed variation in the taxa composition across the burial microcosms during decomposition, with Firmicutes being the dominant phylum, followed by Proteobacteria. At the genus level, *Bacillus* and *Clostridium* were the main genera within Firmicutes. Functional prediction revealed that the most abundant Kyoto encyclopedia of genes and genomes metabolic functions were carbohydrate and amino acid metabolisms.

**Conclusion:**

This study demonstrated a higher bacteria diversity in UA and UAn microcosms than in SA and SAn microcosms. In addition, the taxonomic composition of the microbial community also exhibited changes, highlighting the impact of soil sterilization and oxygen on carcass decomposition. Furthermore, this study provided insights into the microbial communities associated with decomposing swine carcasses in microcosm.

## INTRODUCTION

Outbreaks of contagious animal diseases such as foot-and-mouth disease and African swine fever can lead to significant losses in the livestock industry [1]. Burial is a commonly used method for disposing of both daily and disease-related animal mortalities [2]. However, the metabolites from decomposing carcasses may have adverse effects on the environment, such as soil and groundwater pollution, as well as posing a risk to human and animal health [3]. The decomposition of carcasses can cause dynamic changes in the bacterial communities in the soil, which can adversely affect the environment and lead to possible disease outbreak.

During a disease outbreak, proper disposal of animals associated with infectious pathogens should be implemented to minimize the risk of disease spreading. The disinfection of burial pits for contagious disease-related animal mortalities is usually performed to reduce the spread of disease or reduce the contamination in the environment [4]. Some methods for soil sterilization are through the use of chemicals or heat which can be effective in killing off the microorganisms in the soil [5]. Owing to the heat generated during sterilization, there are decreases in microbial biomass and enzyme activity, resulting in the inactivation of enzymes released by soil microorganisms. The decomposition of buried carcasses mostly relies on the capacity of microbes to generate extracellular proteolytic enzymes, which aid in the breakdown of complex organic matter polymers into smaller oligomeric and monomeric molecules [6]. The rate of carcass decomposition is significantly influenced by microbial activity both within, on, and around the carcasses, as it contributes to the maintenance of soil quality through its involvement in organic matter dynamics, nutrient cycling, and decomposition [7]. In addition, various biotic and abiotic factors can influence the carcass and can cause an adverse effect in the soil microbiome [8].

Aside from the sterilization of soil, the availability of oxygen can affect decomposition and contribute to the changes in the microbial community during carcass decomposition. Various studies have shown that decomposition typically occurs at a faster rate under aerobic conditions [9], while others report faster decomposition under anaerobic conditions [10]. The microbial communities involved in the decomposition of animal carcasses may vary depending on whether the animals were buried or left to decompose naturally in the environment; thus, different aerobic and anaerobic bacteria may be involved in the decomposition of animal carcasses.

Several studies have been conducted to characterize the microbial community composition in decomposing carcasses. However, limited research has been conducted on the microbial community structure of swine carcasses in soil, with or without indigenous microbial communities, during aerobic or anaerobic decomposition. These factors may contribute to the changes in the microbial community composition in decomposing carcasses. Therefore, it is necessary to investigate the changes in the bacterial community in animal burial soil. Thus, the present study focused on the investigation of the changes in the composition and functional diversity of bacterial communities of decomposing swine carcasses in a burial microcosm under the influence of various conditions: carcasses buried in either i) unsterilized soil (soil with an intact microbial community) or ii) soil that was sterilized and was incubated either aerobically or anaerobically.

## MATERIALS AND METHODS

### Ethical statement

This study was conducted in accordance with the guidelines and regulations set by the Institutional Animal Care and Use Committee (Approval number: SCNU IACUC-2019-7) of Sunchon National University (Suncheon, Korea). All experimental protocols were approved by the aforementioned governing body.

### Soil preparation

Soil (10 kg) was collected from an agricultural field at the experimental farm of the Sunchon National University. The raw soil comprised sandy loam soil and had a pH (1:5H_2_O) of 5.84 and a moisture content of 23.70%. The soil was sieved (2 mm) and split into two portions. One portion of the collected soil was sterilized, while the other was kept unsterilized. The sterilized soil was prepared by autoclaving the soil (121°C at 15 psi for 30 minutes) thrice over 4 days to eliminate microbes, fungi, and their spores [5].

### Pre-processing of carcass

A young domestic pig (*Sus scrofa* L.), weighing 10.0±2.0 kg, was purchased commercially and used in the study. The swine was sacrificed, and carcasses were preprocessed prior to decomposition. The carcass was carefully separated from the bones, and the blood and internal organs were collected. Following bone removal, the carcass, skin, blood, and internal organs were homogenized in a mixer and used for the laboratory microcosm to simulate the decomposition process.

### Preparation of microcosm for carcass decomposition

A laboratory microcosm was prepared for the decomposition of the swine carcass. The burial microcosms used two types of soil (soil with intact microbes (unsterilized soil)); ii. soil that was sterilized and two incubation conditions (aerobic and anaerobic conditions). The experimental microcosms were: UA, unsterilized soil – aerobic condition; SA, sterilized soil – aerobic condition; UAn, unsterilized soil – anaerobic condition; and San, sterilized soil – anaerobic condition (Supplementary Figure S1). Soil (112.5 g) and homogenized carcass (37.5 g) were mixed thoroughly and distributed into sterile containers (dimensions: 109 mm×152 mm×58 mm). The soil/carcass ratio depicts the heavy burial conditions of an estimated 550 pig carcasses in a 100 m^2^ burial area [11]. The anaerobic condition was achieved by sealing the container and placing it in a 5% CO_2_ incubator, while for aerobic conditions, the lid of the container was pierced to allow air to pass through the hole before placing it in the incubator. All experimental setups were conducted in triplicate and incubated at 25°C for a total of 60 days.

### Sample collection

Approximately 10 g of the carcass-soil mixture samples were collected from the microcosms at the initial placement time and after 5, 10, 30, and 60 days of decomposition. The samples were placed in sterile conical tubes and kept at −80°C until used.

### Library construction and amplicon sequencing

The carcass-soil samples were sent to Macrogen Inc. (Seoul, Korea) for metagenomic sequencing analysis. The bacterial communities were characterized by analyzing the V3–V4 region of the 16S rRNA gene according to the 16S Metagenomics Library Prep Guide (15044223 Rev. B) [12]. Paired-end sequencing was performed on a MiSeq platform (Illumina, San Diego, CA, USA) using v3 reagents at Macrogen Inc. (Korea).

### Bioinformatics and data analyses

Following sequencing, the raw data was classified by sample using an index sequence, and paired-end FASTQ files were generated for each sample. Subsequently, the raw sequences were demultiplexed, and barcodes and adaptors sequence were removed using the Cutadapt v3.2 program [13]. Sequence reads were clustered into amplicon sequence variants (ASVs) according to the standard pipeline workflow of Divisive Amplicon Denoising Algorithm 2 (DADA2) v1.18.0 [14]. For the paired-end reads, forward and reverse reads were truncated at 250 bp and 200 bp, respectively, and sequences with expected errors of ≥2 were excluded. The QIIME v1.9 program was used for the comparative analysis of the microbial community [15]. Each of the DNA sequences was annotated to the species level using BLAST+ (v.2.9.0) against the Reference Database (NCBI 16S Microbial DB) [16].

Data analysis and visualization were conducted using the MicrobiomeAnalyst web-based tool [17,18]. The processed sequence data were imported into MicrobiomeAnalyst and filtered for low count and low variance using the default settings. This resulted in the removal of 631 low abundance features based on prevalence and 39 low variance features based on inter-quantile range). Subsequently, data normalization was performed using ‘total sum scaling’ as the scaling method. Shannon’s diversity index and Chao1 richness were calculated and used to compare the alpha diversity in the microcosms. Statistical analysis was performed using the general linear model procedure of the Statistical Analysis System (SAS) program version 9.4 (SAS Institute Inc., Cary, NC, USA). Two-way analysis of variance was used to examine the effects of the presence or absence of soil microbes, oxygen availability, and their interaction in the alpha diversity indices. Tukey’s honestly significant difference post hoc test was used to compare significant differences between burial microcosms at each time point, with a significance level set at p<0.05. Beta diversity at the genus level was assessed based on the Bray-Curtis distance method, and the results were visualized using principal coordinate analysis (PCoA). Bacterial abundance profiles at the phylum, genus, and species levels were represented using stacked bar graphs. Venn diagram of unique and core bacterial genera was drawn using jvenn [19] to highlight the similarities and shared sequences between the different microcosms. Analysis of the core microbiome was carried out at the genus level using the MicrobiomeAnalyst with sample prevalence at 20% and a relative abundance cutoff of 0.01%.

### Prediction of functional profile of bacterial communities in decomposing swine carcass

MicrobiomeAnalyst was employed to predict the functional profiles of bacterial communities associated with decomposing swine carcasses based on 16S rRNA gene sequencing data [17,18]. The ASV table and metadata table were uploaded to the Marker Data Profiling Module. Functional profiles were determined by analyzing the ASVs using Tax4Fun in MicrobiomeAnalyst. The resulting Kyoto encyclopedia of genes and genomes (KEGG) Orthology (KO) table [20] was then imported to the Shotgun Data Profiling Module, and diversity and association analyses were investigated.

## RESULTS

### Bacterial species richness and diversity

**Figure 1:**
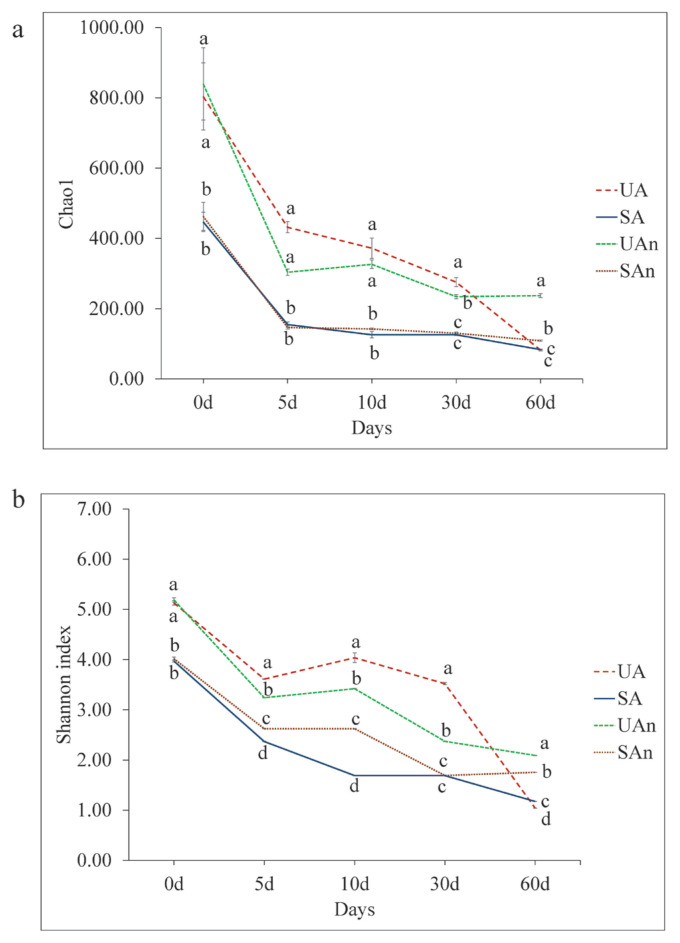
Alpha diversity metrics of the bacterial community of decomposing swine carcasses in different burial microcosms at each time point: (a) Chao1 and (b) Shannon diversity index. The error bar represents the standard error of the mean. UA, unsterilized soil–aerobic condition; SA, sterilized soil–aerobic condition; UAn, unsterilized soil–anaerobic condition; and SAn, sterilized soil–anaerobic condition. Superscripts (^a–d^) indicate significant differences among burial microcosms at each time point according to Tukey honestly significant difference test at p<0.05.

The alpha diversity represented by Chao1 and Shannon’s diversity indices for the different microcosms is shown in Figure 1. Chao1 showed significant differences among burial microcosms (p<0.05) in all time points (Figure 1a). Chao1 was significantly higher (p<0.05) in UA and UAn microcosms than in SA and SAn microcosms during the initial placement, then decreased throughout the decomposition period. At the end of the study period, lowest Chao1 were observed in UA, SA, and SAn microcosms. The Shannon’s diversity index showed significant differences among the microcosms in all periods (p<0.05) (Figure 1b). At day 0, Shannon index in UA and UAn were significantly higher compared to SA and SAn microcosms. At days 5, 10, 30, and 60, Shannon index was significantly different between treatments (p<0.05). Moreover, the Shannon’s diversity index in the UA and UAn microcosms were higher than those in SA and SAn microcosms until day 30. At day 60, Shannon’s diversity index were higher in UAn and SAn than in UA and SA microcosms (p<0.05).

**Figure 2:**
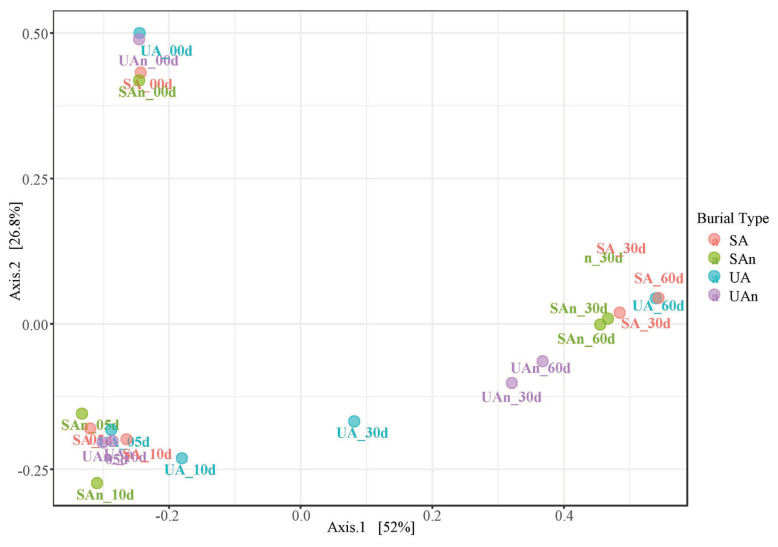
Principal coordinate analysis (PCoA) plot showing the variation in bacterial communities among microcosms. SA, sterilized soil–aerobic condition; SAn, sterilized soil–anaerobic condition; UA, unsterilized soil–aerobic condition; UAn, unsterilized soil–anaerobic condition.

Comparisons of the microcosms at different time points using PCoA revealed that the samples at day 0 showed a well-differentiated bacterial profile. Moreover, axis 1 and axis 2 explained 52% and 26.9% of the variance, respectively. The first two principal components (Axis1+Axis2) accounted for 78.9% of the total variation (Figure 2). Microcosm samples on days 5 and 10 were grouped together and at different distances from the other time points. In addition, at the beginning of decomposition (day 0) and after the late stages (days 30 and 60), samples were found dispersed in axes, indicating a differentiated bacterial community based on 16S rRNA amplicon sequencing.

### Core microbiome

**Figure 3:**
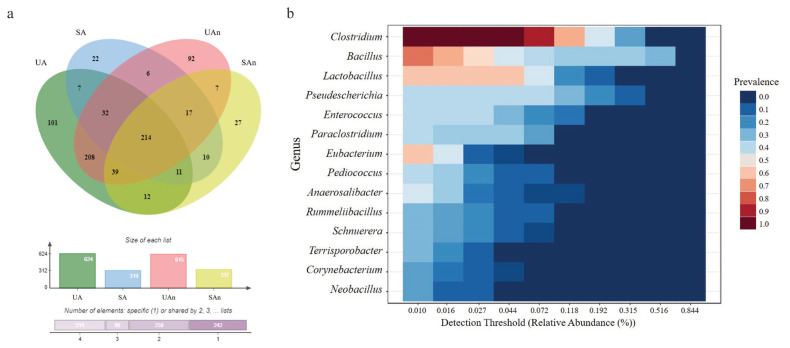
Bacterial core microbiome in the swine burial microcosms. Venn diagram of shared and unique bacterial species among different swine burial microcosms (a). Core microbiome at the genus level in all the swine microcosms (b). UA, unsterilized soil–aerobic condition; SA, sterilized soil–aerobic condition; UAn, unsterilized soil–anaerobic condition; and SAn, sterilized soil–anaerobic condition.

In total, 1,687 ASVs were detected in all soil microcosm samples, of which 22 phyla, 805 genera, and 1,687 species were identified across all samples. The Venn diagram represents the shared bacterial species within all microcosms, as well as the unique species within the different samples (Figure 3a). Results showed that 101, 22, 92, and 27 genera were uniquely present in UA, SA, UAn, and SAn, respectively (Supplementary Table S1). We found 7 unique bacterial genera (*Alistipes*, *Natranaerovigra*, *Acinetobacter*, *Ralstonia*, *Achromobacter*, *Novosphingobium*, and *Comomonas*) between UA and SA (Supplementary Table S2). In addition, 7 unique bacterial genera (*Geothermomicrobium*, *Ramlibacter*, *Jeotgalibacillus*, *Allisonella*, *Rothia*, *Thermobacillus*, and N*eglecta*) were identified between UAn and SAn. Meanwhile, 208 and 10 unique genera were found between UA and UAn microcosms and SA and SAn microcosms, respectively. Moreover, we found 214 genera were shared among all four microcosms (Supplementary Table Table S3). Although 805 genera were identified in the analyzed samples, 14 genera, *Clostridium*, *Bacillus*, *Lactobacillus*, *Pseudescherichia*, *Enterococcus*, *Paraclostridium*, *Eubacterium*, *Pediococcus*, *Anaerosalibacter*, *Rummeliibacillus*, *Schnuerera*, T*errisporobacter*, *Corynebacterium*, and *Neobacillus*, constituted the core microbiome of the analyzed samples from the swine burial microcosms (Figure 3b).

### Bacterial community composition during decomposition

**Figure 4:**
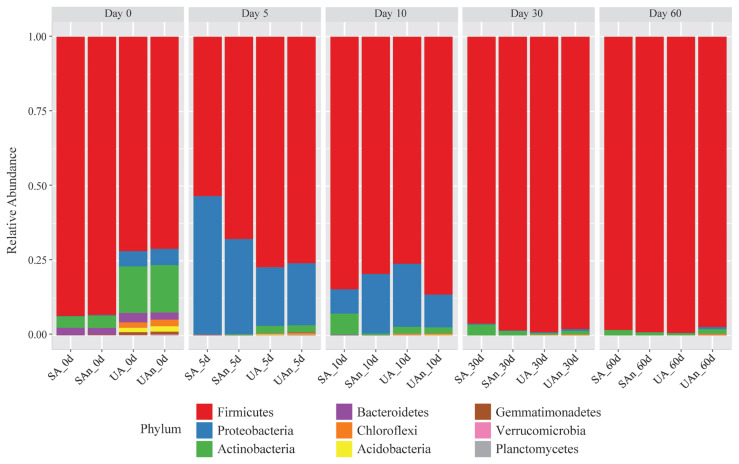
Relative abundance of bacterial communities at phylum level in different microcosms. SA, sterilized soil–aerobic condition; SAn, sterilized soil–anaerobic condition; UA, unsterilized soil–aerobic condition; UAn, unsterilized soil–anaerobic condition.

The taxonomic composition of each microcosm was analyzed and compared at the phylum, genus, and species levels. The dominant taxa varied among the microcosms at different time points. Evaluation of the bacterial ASVs revealed that Firmicutes was the major phylum identified in all the microcosms, followed by Proteobacteria and Actinobacteria (Figure 4).

Classification of the identified bacterial ASVs revealed that during the initial day (day 0), Firmicutes was the most dominant phylum in the burial microcosm, which represented 57.62%, 90.68%, 57.43%, and 90.30% of the relative abundances for UA, SA, UAn, and SAn microcosms, respectively. Actinobacteria was the next most abundant phylum in the UA (17.92%) and UAn (17.84%) microcosms. On day 5, the relative abundance of Firmicutes increased in UA (79.98%) and UAn (75.39%), whereas the abundance was reduced in SA (53.66%) and SAn (68%). Proteobacteria increased in abundance in all samples, particularly in SA (45.89%), and SAn (31.52%). After 10 days, the relative abundance of Proteobacteria was reduced in all samples, more particularly in SA and SAn, where an apparent reduction in abundance was observed. Moreover, Firmicutes increased in abundance in all samples and became the most abundant phylum at this point. On subsequent days (days 30 and 60), a clear domination of the members of the phylum Firmicutes, which represented 96% to 99% of the population in all samples was observed, whereas the abundance of other bacterial phyla decreased.

**Figure 5:**
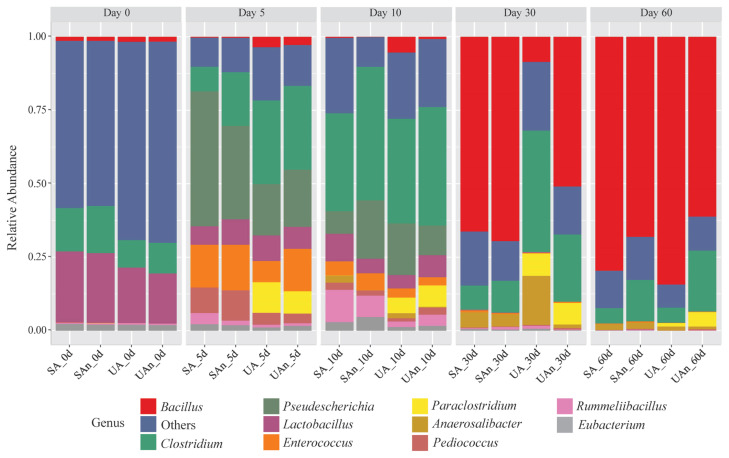
Relative abundance of bacterial communities at genus level in different microcosms. SA, sterilized soil–aerobic condition; SAn, sterilized soil–anaerobic condition; UA, unsterilized soil–aerobic condition; UAn, unsterilized soil–anaerobic condition.

As shown in Figure 5, *Bacillus*, *Clostridium*, *Enterococcus*, and *Lactobacillus* were among the major bacterial genera in the microcosms. The abundances of these bacterial genera shifted throughout the decomposition period. On day 0, *Lactobacillus* and *Clostridium* were the most dominant genera in the samples; however, a higher abundance was observed in microcosms SA and SAn than what was seen in UA and UAn. After 5 days, *Clostridium*, *Pseudescherichia*, *Enterococcus*, *Paraclostridium*, and *Pediococcus* were among the genera that increased in abundance. Moreover, the abundance of *Pseudescherichia* sharply increased in all the samples, particularly in the SA and SAn. In addition, Clostridium and Paraclostridium increased in abundance in the UA and UAn microcosms. In contrast, *Enterococcus* abundance increased in all microcosms, particularly in SA, UAn, and SAn. On day 10, *Clostridium* increased in all samples and was identified as the most dominant genus. A higher abundance of *Clostridium* was observed in the UAn and SAn microcosms. Meanwhile, the relative abundances of *Pseudescherichia*, *Paraclostridium*, and *Enterococcus* were reduced in all samples, whereas the abundance of *Rummeliibacillus* increased, particularly in the microcosms SA and SAn. On day 30, members of *Bacillus* dominated UAn, SA, and SAn at 65.84%, 50.75%, and 69.24%, respectively. Consequently, a sharp decline in the abundance of *Clostridium* was observed in UAn, SA, and SAn, whereas the abundance in UA was maintained. In addition, *Anaerosalibacter* was detected in UA at an abundance of 12.73% which was higher than that in other burial microcosms. On day 60, members of the *Bacillus* continued to be dominant in all samples. Moreover, a higher abundance of *Bacillus* was observed in microcosms UA and SA than in UAn and SAn.

**Figure 6:**
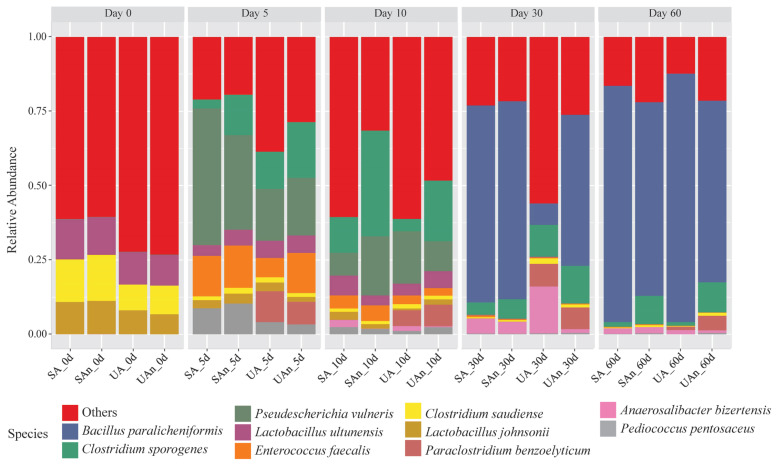
Relative abundance of bacterial communities at species level in different microcosms. SA, sterilized soil–aerobic condition; SAn, sterilized soil–anaerobic condition; UA, unsterilized soil–aerobic condition; UAn, unsterilized soil–anaerobic condition.

The major bacterial species in each burial microcosm are shown in Figure 6. The composition of bacterial species varied in each burial microcosm and at each time point. On the initial day, *Lactobacillus ultunensis*, *L. johnsonii*, and *Clostridium saudiense* were the most abundant species in all samples. However, higher abundances of these species were found in the SA and SAn microcosms compared to the UA and UAn microcosms. By day 5, *Pseudescherichia vulneris*, *Clostridium sporogenes*, *Enterococcus faecalis*, *Paraclostridium benzoelyticium*, and *Pediococcus pentosaceus* were among the dominant genera observed in the samples. Specifically, *P. vulneris* was more abundant in SA and SAn, while *C. sporogenes* was more abundant in UA, UAn, and SAn, and *E. faecalis* was abundant in SA, UAn, and SAn. In addition, the relative abundance of *P. pentosaceus* increased in all the samples. We also identified *P. benzoelyticium* in UA and UAn microcosms. By day 10, *C. sporogenes* decreased in abundance in UA but increased in SA, UAn, and SAn. In particular, the abundance in UAn and SAn was high, with a relative abundance of 20.06% and 35.28%, respectively. Meanwhile, the relative abundances of *P. vulneris*, *P. benzoelyticium*, *E. faecalis*, and *P. pentosaceus* was reduced. On day 30, a clear dominance of *Bacillus paralicheniformis* was noted in the microcosms of SA, UAn, and SAn. *Anaerosalibacter bizertensis* was detected in the UA microcosm with an abundance of 12.73%. By day 60, the abundance of *B. paralicheniformis* increased in all samples and was the most dominant genus at this point, reaching a relative abundance of approximately 59.99% to 82.98% at the end of the experiment.

### Prediction of the functional profile of bacterial communities associated with decomposing swine carcass

**Figure 7:**
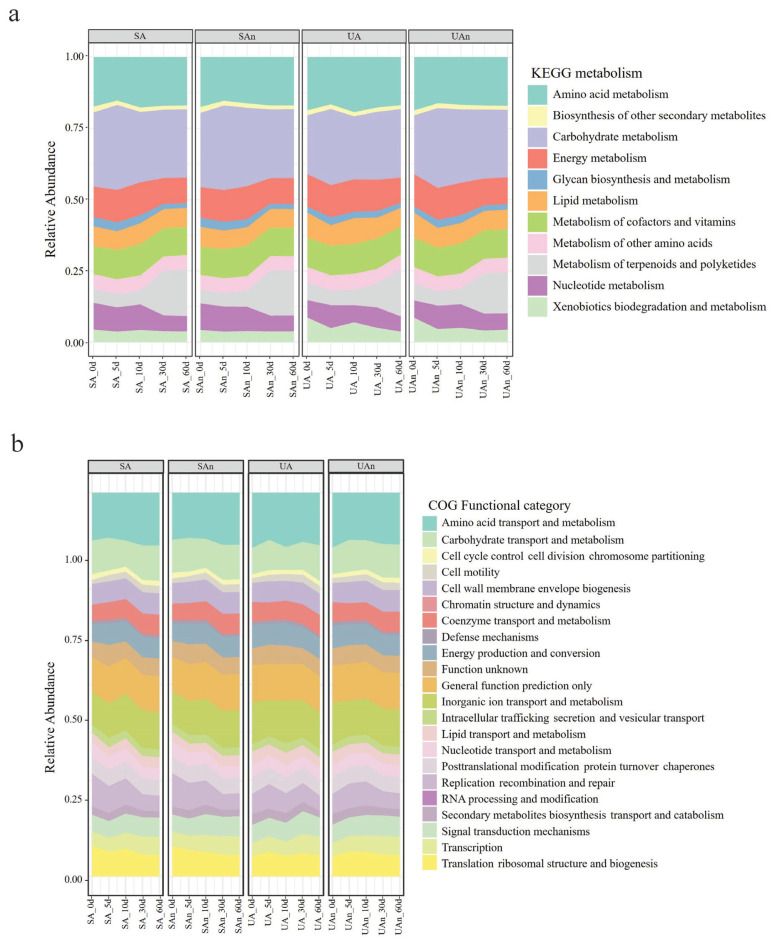
Prediction of functional profile of bacterial communities in decomposing swine carcasses. Kyoto encyclopedia of genes and genomes (KEGG) metabolism (a) and clusters of orthologous groups of proteins (COG) functional categories (b). SA, sterilized soil–aerobic condition; SAn, sterilized soil–anaerobic condition; UA, unsterilized soil–aerobic condition; UAn, unsterilized soil–anaerobic condition.

The probable functions of the decomposition microbiome were inspected by Tax4Fun in the MicrobiomeAnalyst tool. Diversity analysis revealed 5,418 KEGG orthologs, 1,230 KEGG pathways, and 11 KEGG metabolic functions in decomposing swine carcasses. The most abundant KEGG metabolic functions were carbohydrate and amino acid metabolisms (Figure 7a). There were 22 predicted clusters of orthologous groups of proteins (COG) in all microcosms and the most abundant COG were amino acid transport and metabolism, followed by inorganic ion transport and metabolism, and carbohydrate transport and metabolism (Figure 7b).

## DISCUSSION

Microbes play a crucial role in decomposition, as they produce degradative enzymes and can utilize a diverse range of carrion substrates, including internal tissues, organs, skin, hair, and even bone. Therefore, identifying the decomposition ecology in swine microcosms is crucial to strengthen the current knowledge of the microbiology of decomposing carcasses. Previous research on decomposing swine and mice has revealed that bacterial communities undergo changes in major phyla over time, which align with specific visual indicators of body decomposition [21,22]. The variances in the microbial composition observed in our study could potentially be attributed to changes in dominant phyla. Lauber et al [5] revealed that the presence of soil microbial communities has a substantial impact on accelerating the rates of carrion decomposition. Our findings showed that Chao1 index was comparable between UA and UAn microcosms and in SA and SAn microcosms, particularly at day 0 to 10. At days 30 and 60, Chao1 showed variations between different microcosms, with UAn being the highest. Moreover, Shannon’s diversity index showed that variations in bacterial composition was observed among the different microcosms in all periods. These results suggest that the removal of the indigenous microbes in the soil and the oxygen availability during decomposition influenced the changes in the bacterial composition. Moreover, the changes in the bacterial communities suggest that various bacterial species may have played a role during decomposition.

Firmicutes, Proteobacteria, and Actinobacteria were the predominant bacterial phyla in the microcosms. This is consistent with other studies that have reported similar findings regardless of the type of carcass [5,23]. Interestingly, Firmicutes were found to increase in abundance in all microcosms as the decomposition process advanced over time, particularly at days 30 and 60 of decomposition. Several studies also reported the replacement of Proteobacteria by Firmicutes as the dominant phylum during the later stages of decay in swine models [3,23]. Firmicutes are known to be actively involved in the degradation of large macromolecules such as proteins, complex fats, and polycarbohydrates into their constituent building blocks [24]. Additionally, members of Firmicutes are facultative anaerobes or anaerobes, which can thrive in environments with limited oxygen availability and can compete over other bacteria that are less adapted to low oxygen environments. Furthermore, Firmicutes are often among the first groups of bacteria to colonize and initiate the decomposition process in organic matter. Their ability to quickly establish a presence and initiate degradation is advantageous in resource-rich environments such as carcasses, where there is an abundant supply of organic matter. Thus, Firmicutes tend to be more abundant during decomposition processes. The taxa belonging to Proteobacteria are often linked to meat spoilage and have been detected on the skin of slaughtered animals [23]. Additionally, Proteobacteria are commonly found in soil and play a significant role in the decomposition of fats and carbohydrates [25].

The genera *Clostridium*, *Bacillus*, and *Lactobacillus* were the most prevalent core microbes identified from all the swine burial microcosms in the study. The detection of the core microbiota from the microcosms suggests that these bacteria are associated with carcass decomposition. These genera were predominantly present in decomposing carcasses [7,25]. This study indicated notable variations in the genera during decomposition. During the initial day, *Lactobacillus* was found to be more abundant in SA and SAn microcosms than in UA and UAn microcosms. The dominance of these bacteria was due to being part of the gut microflora of the animal [26]. *Lactobacillus* spp. are known to be involved in the breakdown of lipids and complex carbohydrates in animal carcasses [27]. On day 5 of decomposition, the abundance of *Enterococcus* increased, whereas that of *Lactobacillus* decreased in all microcosms. Similarly, Li et al [28] reported that during the early stage of decomposition, gas accumulation caused bloating and rupture of the carcass, leading to a shift from internal to external conditions. This shift resulted in a decrease in anaerobe bacteria like *Lactobacillus*, while the facultative anaerobe *Enterococcus* took advantage of the changed conditions and thrived. In addition, Iancu et al [29] also reported an increase in the abundance of *E. faecalis*, whereas Hauther et al [30] reported a decrease in the abundance of members of *Lactobacillus*. *E. faecalis* is commonly found in human and animal gastrointestinal tracts and can ferment glucose and catabolize carbohydrates, diamino acids, and glycerol [29]. Our findings showed a notable increase in the abundance of *Bacillus* towards the end of the incubation period in all microcosms. *Bacillus* spp. are microorganisms associated with adipocyte decomposition and capable of denitrification [25]. Furthermore, they are known to produce a wide range of non-peptide and peptide antimicrobial compounds that effectively inhibit the growth of other bacteria [31]. The increased abundance of *Bacillus* towards the later stage of decomposition may be attributed to the synergistic or antagonistic interactions between *Bacillus* and other bacteria, which led to the alteration of the microbial community structure. A high abundance of *B. paralicheniformis* was identified in all microcosms, particularly in microcosms under aerobic conditions, suggesting that this bacterium may positively be associated with swine carcass decomposition.

The bacterial community in the microcosms was dominated by *Clostridium*, particularly in UA, UAn, and SAn microcosms. *Clostridium* spp. are part of the normal gut microflora and are anaerobic organisms, but several species may survive in the presence of a small amount of oxygen [32]; therefore, these factors were likely the reason for this high abundance in these microcosms. Members of *Clostridium* spp. are known to play a crucial role in biomass breakdown, as they synthesize a wide variety of extracellular enzymes that aid in the degradation of various compounds, such as carbohydrates, lipids, amino acids, alcohols, and purines [33]. Additionally, several studies have highlighted the significant role of *Clostridium* spp. in carcass decomposition, as they can make up to 20% of the postmortem microbiome and possess proteolytic ability, fast growth rate, and anaerobic capabilities, making them well-suited for decomposing carcasses [34]. Similarly, our findings revealed an abundance of approximately 20% for this genus. Among these species, *C. saudiense* and *C. sporogenes* were identified in the microcosms. The abundance of *C. sporogenes* was substantially higher on days 5, 10, and 60 in the SAn and UAn microcosms, whereas *C. saudiense* was more abundant on day 0 and decreased in abundance as decomposition progressed in the SA and SAn microcosms. It has been reported that *C. sporogenes* was one of the most abundant species during decomposition [28].

The microbiota associated with carcass decomposition demonstrated diverse functional pathways. This diversity reflects the potential roles of microbes as decomposers. We detected expected increases in the expression of genes related to carbohydrate and amino acid metabolism. The up-regulation of carbohydrate and amino acid metabolism suggests that nutritional utilization plays a crucial role in determining which species become dominant [35]. Furthermore, Firmicutes have been reported to ferment amino acids and peptides into propionate and butyrate, which can contribute to the production of odor. Moreover, the up-regulation of carbohydrate metabolism is associated with increases in concentrations of hydrogen, carbon dioxide, hydrogen sulfide, and methane during decomposition [36]. On day 5, we observed an upregulation in the expression of genes related to carbohydrate metabolism and amino acid metabolism, which subsequently decreased on day 10. These findings indicate that the degradation of amino acids and carbohydrates within the carcasses decreased, likely due to the release of nutrient-rich fluids into the surrounding environment [37].

Several studies have focused on the quantification and identification of bacterial species associated with decomposition. Identifying the changes in bacterial community is significant for further understanding the decomposition microbiome. It is also important to note that laboratory microcosm experiments are just one tool for investigating the complex processes of decomposition in soil, and their results may not always be directly applicable to natural settings. Nonetheless, such experiments can provide valuable insights into the underlying changes in the microbial community during decomposition and help inform our understanding of the ecological and environmental impacts of animal carcass disposal. Overall, our findings provide microbiome information on carcasses decomposed in soil with or without microbes under different conditions of oxygen availability. The results of the present study are beneficial for estimating the microbes associated with the decomposition of swine carcasses. However, quantitative differences must be expected as each carcass has its unique microbiome composition.

## CONCLUSION

This study evaluated the changes in the bacterial communities of different microcosms of decomposing swine carcasses over a 60 day period. Our findings demonstrated the composition of the bacterial communities was significantly influenced by factors such as soil sterilization and oxygen availability. In particular, Chao1 and Shannon diversity indices were significantly higher in UA and UAn compared to SA and SAn at the start of the experiment; however, diversity indices decreased over time. Variations in the bacterial taxonomic composition between UA and UAn to SA and SAn microcosms were observed throughout the decomposition period, suggesting that the removal of indigenous microbes in the soil and oxygen availability during decomposition influenced the shifts in the microbial composition. In addition, we detected predicted functional genes associated with the decomposition of carcasses. These findings provide valuable insights into the underlying changes in the diversity, structure, and composition of bacterial communities in swine carcasses decomposed under different conditions *in vitro*. Despite these findings, the association and functional role of these bacterial species on carcass decomposition is limited; thus, further research is necessary in this field.

## SUPPLEMENTARY MATERIAL

**Supplementary Figure S1**. Illustration of the incubation boxes for aerobic and anaerobic conditions.

**Supplementary Table S1**. Taxonomic classification of bacterial genera present only in UA, SA, UAn, and SAn samples

**Supplementary Table S2**. Taxonomic classification of shared bacterial genera present between UA, SA, UAn, and SAn samples

**Supplementary Table S3**. Taxonomic classification of shared bacterial genera present in UA, SA, UAn, and SAn samples
