# Supplementary figures and images for: Composition and functional diversity of bacterial communities during swine carcass decomposition
Source: Anim Biosci. 2023 Jun 26;36(9):1453–64. doi: 10.5713/ab.23.0140 (PMC10472150; doi:10.5713/ab.23.0140)

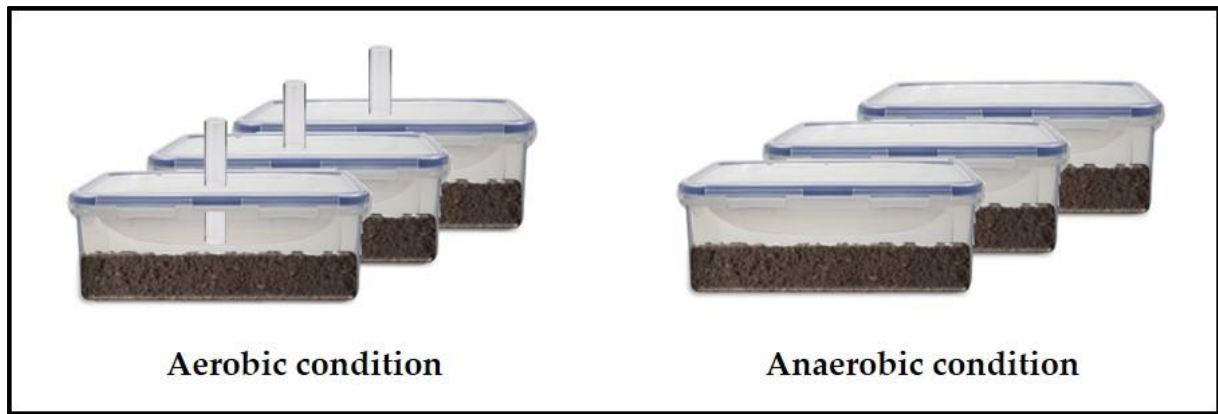

**Figure S1.** Illustration of the incubation boxes for aerobic and anaerobic conditions.

Supplement: Supplementary file 1 [file ab-23-0140-Supplementary-Fig-1.pdf]
