# Supplementary material for: Composition and functional diversity of bacterial communities during swine carcass decomposition
Source: Anim Biosci. 2023 Jun 26;36(9):1453–64. doi: 10.5713/ab.23.0140 (PMC10472150; doi:10.5713/ab.23.0140)
Supplement: Supplementary file 2 [file ab-23-0140-Supplementary-Table-1.pdf]

Table S1. Taxonomic classification of bacterial genera present only in UA, SA, UAn, and SAn samples

Taxonomy	Burial microcosm
Bacteria; Actinobacteria; Actinomycetia; Micrococcales; Ruaniaceae; Ruania	UA
Bacteria; Firmicutes; Clostridia; Thermoanaerobacterales; Thermoanaerobacteraceae; Desulfovirgula	UA
Bacteria; Actinobacteria; Actinomycetia; Micrococcales; Unclassified Micrococcales; Luteimicrobium	UA
Bacteria; Firmicutes; Bacilli; Bacillales; Bacillaceae; Fredinandcohnia	UA
Bacteria; Bacteroidetes; Cytophagia; Cytophagales; Hymenobacteraceae; Pontibacter	UA
Bacteria; Actinobacteria; Actinomycetia; Micrococcales; Micrococcaceae; Glutamicibacter	UA
Bacteria; Proteobacteria; Gammaproteobacteria; Xanthomonadales; Xanthomonadaceae; Thermomonas	UA
Bacteria; Bacteroidetes; Sphingobacteriia; Sphingobacteriales; Sphingobacteriaceae; Sphingobacterium	UA
Bacteria; Acidobacteria; Acidobacteriia; Acidobacteriales; Acidobacteriaceae; Acidipila	UA
Bacteria; Proteobacteria; Betaproteobacteria; Burkholderiales; Alcaligenaceae; Alcaligenes	UA
Bacteria; Proteobacteria; Deltaproteobacteria; Myxococcales; Polyangiaceae; Polyangium	UA
Bacteria; Proteobacteria; Gammaproteobacteria; Enterobacterales; Morganellaceae; Providencia	UA
Bacteria; Actinobacteria; Actinomycetia; Pseudonocardiales; Pseudonocardiaceae; Actinokineospora	UA
Bacteria; Proteobacteria; Alphaproteobacteria; Rhodospirillales; Rhodospirillaceae; Rhodovibrio	UA
Bacteria; Actinobacteria; Actinomycetia; Streptosporangiales; Nocardiopsaceae; Nocardiopsis	UA
Bacteria; Proteobacteria; Alphaproteobacteria; Rhodospirillales; Rhodospirillaceae; Ferrovibrio	UA
Bacteria; Proteobacteria; Gammaproteobacteria; Xanthomonadales; Xanthomonadaceae; Stenotrophomonas	UA
Bacteria; Proteobacteria; Betaproteobacteria; Burkholderiales; Oxalobacteraceae; Noviherbaspirillum	UA
Bacteria; Firmicutes; Clostridia; Eubacteriales; Lachnospiraceae; Anaerobium	UA
Bacteria; Firmicutes; Clostridia; Eubacteriales; Lachnospiraceae; Acetatifactor	UA
Bacteria; Proteobacteria; Alphaproteobacteria; Hyphomicrobiales; Kaistiaceae; Kaistia	UA
Bacteria; Proteobacteria; Alphaproteobacteria; Caulobacterales; Caulobacteraceae; Asticcacaulis	UA
Bacteria; Firmicutes; Clostridia; Eubacteriales; Heliobacteriaceae; Heliophilum	UA
Bacteria; Proteobacteria; Deltaproteobacteria; Syntrophobacterales; Syntrophobacteraceae; Desulfoglaeba	UA
Bacteria; Proteobacteria; Oligoflexia; Oligoflexales; Oligoflexaceae; Oligoflexus	UA
Bacteria; Bacteroidetes; Flavobacteriia; Flavobacteriales; Flavobacteriaceae; Myroides	UA
Bacteria; Actinobacteria; Actinomycetia; Geodermatophilales; Geodermatophilaceae; Cumulibacter	UA
Bacteria; Proteobacteria; Alphaproteobacteria; Caulobacterales; Caulobacteraceae; Caulobacter	UA
Bacteria; Firmicutes; Clostridia; Eubacteriales; Clostridiaceae; Natronincola	UA
Bacteria; Proteobacteria; Deltaproteobacteria; Desulfuromonadales; Geobacteraceae; Geothermobacter	UA
Bacteria; Firmicutes; Clostridia; Eubacteriales; Lachnospiraceae; Lachnotalea	UA
Bacteria; Proteobacteria; Gammaproteobacteria; Methylococcales; Methylococcaceae; Methylocaldum	UA
Bacteria; Firmicutes; Clostridia; Eubacteriales; Oscillospiraceae; Pseudoflavonifractor	UA
Bacteria; Actinobacteria; Actinomycetia; Streptosporangiales; Streptosporangiaceae; Sphaerimonospora	UA
Bacteria; Acidobacteria; Thermoanaerobaculia; Thermoanaerobaculales; Thermoanaerobaculaceae; Thermoanaerobaculum	UA
Bacteria; Proteobacteria; Gammaproteobacteria; Enterobacterales; Enterobacteriaceae; Pseudocitrobacter	UA
Bacteria; Actinobacteria; Actinomycetia; Micromonosporales; Micromonosporaceae; Salinispora	UA
Bacteria; Proteobacteria; Gammaproteobacteria; Methylococcales; Methylococcaceae; Methylococcus	UA
Bacteria; Firmicutes; Clostridia; Eubacteriales; Oscillospiraceae; Hydrogenoanaerobacterium	UA
Bacteria; Proteobacteria; Gammaproteobacteria; Oceanospirillales; Oceanospirillaceae; Nitrincola	UA
Bacteria; Proteobacteria; Alphaproteobacteria; Hyphomicrobiales; Hyphomicrobiaceae; Filomicrobium	UA
Bacteria; Firmicutes; Clostridia; Eubacteriales; Oscillospiraceae; Saccharofermentans	UA
Bacteria; Bacteroidetes; Chitinophagia; Chitinophagales; Chitinophagaceae; Taibaiella	UA
Bacteria; Firmicutes; Bacilli; Bacillales; Unclassified Bacillales; Desulfuribacillus	UA
Bacteria; Actinobacteria; Actinomycetia; Micrococcales; Intrasporangiaceae; Knoellia	UA
Bacteria; Proteobacteria; Gammaproteobacteria; Alteromonadales; Alteromonadaceae; Mangrovitalea	UA
Bacteria; Proteobacteria; Alphaproteobacteria; Caulobacterales; Caulobacteraceae; Brevundimonas	UA
Bacteria; Proteobacteria; Alphaproteobacteria; Hyphomicrobiales; Phyllobacteriaceae; Aminobacter	UA
Bacteria; Bacteroidetes; Bacteroidia; Bacteroidales; Dysgonomonadaceae; Fermentimonas	UA
Bacteria; Actinobacteria; Actinomycetia; Micrococcales; Ornithinimicrobiaceae; Ornithinimicrobium	UA
Bacteria; Actinobacteria; Actinomycetia; Micromonosporales; Micromonosporaceae; Luedemannella	UA
Bacteria; Actinobacteria; Actinomycetia; Micrococcales; Jonesiaceae; Flavimobilis	UA
Bacteria; Proteobacteria; Alphaproteobacteria; Hyphomicrobiales; Brucellaceae; Mycoplana	UA
Bacteria; Proteobacteria; Alphaproteobacteria; Rhodospirillales; Acetobacteraceae; Acidisphaera	UA
Bacteria; Actinobacteria; Actinomycetia; Micrococcales; Micrococcaceae; Enteractinococcus	UA
Bacteria; Verrucomicrobia; Verrucomicrobiae; Verrucomicrobiales; Verrucomicrobiaceae; Verrucomicrobium	UA
Bacteria; Firmicutes; Clostridia; Eubacteriales; Defluviitaleaceae; Defluviitalea	UA
Bacteria; Bacteroidetes; Cytophagia; Cytophagales; Cytophagaceae; Leadbetterella	UA
Bacteria; Actinobacteria; Acidimicrobiia; Acidimicrobiales; Iamiaceae; Aquihabitans	UA
Bacteria; Firmicutes; Bacilli; Bacillales; Paenibacillaceae; Gorillibacterium	UA
Bacteria; Cyanobacteria; Unclassified Cyanobacteria; Nostocales; Calotrichaceae; Dulcicalothrix	UA
Bacteria; Proteobacteria; Betaproteobacteria; Burkholderiales; Alcaligenaceae; Castellaniella	UA
Bacteria; Bacteroidetes; Cytophagia; Cytophagales; Fulvivirgaceae; Chryseolinea	UA
Bacteria; Verrucomicrobia; Opitutae; Opitutales; Opitutaceae; Alterococcus	UA
Bacteria; Proteobacteria; Alphaproteobacteria; Rhodospirillales; Geminicoccaceae; Geminicoccus	UA
Bacteria; Proteobacteria; Deltaproteobacteria; Unclassified Deltaproteobacteria; Dissulfurirhabdaceae; Dissulfurirhabdus	UA
Bacteria; Actinobacteria; Actinomycetia; Micrococcales; Microbacteriaceae; Microbacterium	UA
Bacteria; Firmicutes; Clostridia; Eubacteriales; Eubacteriaceae; Acetobacterium	UA
Bacteria; Proteobacteria; Betaproteobacteria; Burkholderiales; Comamonadaceae; Variovorax	UA
Bacteria; Proteobacteria; Betaproteobacteria; Nitrosomonadales; Gallionellaceae; Sideroxydans	UA
Bacteria; Proteobacteria; Gammaproteobacteria; Xanthomonadales; Xanthomonadaceae; Unclassified Xanthomonadaceae	UA
Bacteria; Proteobacteria; Alphaproteobacteria; Hyphomicrobiales; Rhizobiaceae; Sinorhizobium	UA
Bacteria; Firmicutes; Clostridia; Eubacteriales; Oscillospiraceae; Ethanoligenens	UA
Bacteria; Acidobacteria; Acidobacteriia; Acidobacteriales; Acidobacteriaceae; Granulicella	UA

Bacteria; Proteobacteria; Alphaproteobacteria; Rhodospirillales; Rhodospirillaceae; Telmatospirillum	UA
Bacteria; Proteobacteria; Gammaproteobacteria; Chromatiales; Ectothiorhodospiraceae; Alkalilimnicola	UA
Bacteria; Proteobacteria; Deltaproteobacteria; Desulfobacterales; Desulfobacteraceae; Desulfatibacillum	UA
Bacteria; Actinobacteria; Coriobacteriia; Eggerthellales; Eggerthellaceae; Paraeggerthella	UA
Bacteria; Proteobacteria; Alphaproteobacteria; Rhodobacterales; Rhodobacteraceae; Thioclava	UA
Bacteria; Firmicutes; Bacilli; Bacillales; Bacillaceae; Halolactibacillus	UA
Bacteria; Proteobacteria; Epsilonproteobacteria; Campylobacterales; Campylobacteraceae; Campylobacter	UA
Bacteria; Proteobacteria; Deltaproteobacteria; Myxococcales; Myxococcaceae; Pyxidicoccus	UA
Bacteria; Proteobacteria; Gammaproteobacteria; Chromatiales; Ectothiorhodospiraceae; Thioalbus	UA
Bacteria; Proteobacteria; Alphaproteobacteria; Hyphomicrobiales; Beijerinckiaceae; Pseudochelatococcus	UA
Bacteria; Proteobacteria; Alphaproteobacteria; Rhodobacterales; Rhodobacteraceae; Albimonas	UA
Bacteria; Proteobacteria; Betaproteobacteria; Burkholderiales; Comamonadaceae; Diaphorobacter	UA
Bacteria; Proteobacteria; Alphaproteobacteria; Hyphomicrobiales; Phyllobacteriaceae; Pseudaminobacter	UA
Bacteria; Proteobacteria; Alphaproteobacteria; Hyphomicrobiales; Xanthobacteraceae; Starkeya	UA
Bacteria; Proteobacteria; Alphaproteobacteria; Hyphomicrobiales; Brucellaceae; Brucella	UA
Bacteria; Proteobacteria; Betaproteobacteria; Burkholderiales; Alcaligenaceae; Pusillimonas	UA
Bacteria; Firmicutes; Bacilli; Lactobacillales; Carnobacteriaceae; Pisciglobus	UA
Bacteria; Actinobacteria; Actinomycetia; Micrococcales; Bogoriellaceae; Georgenia	UA
Bacteria; Bacteroidetes; Chitinophagia; Chitinophagales; Chitinophagaceae; Chitinophaga	UA
Bacteria; Proteobacteria; Oligoflexia; Bacteriovoracales; Bacteriovoracaceae; Peredibacter	UA
Bacteria; Acidobacteria; Blastocatellia; Blastocatellales; Blastocatellaceae; Aridibacter	UA
Bacteria; Actinobacteria; Actinomycetia; Corynebacteriales; Lawsonellaceae; Lawsonella	UA
Bacteria; Proteobacteria; Deltaproteobacteria; Myxococcales; Myxococcaceae; Aggregicoccus	UA
Bacteria; Firmicutes; Clostridia; Eubacteriales; Peptococcaceae; Thermincola	UA
Bacteria; Proteobacteria; Gammaproteobacteria; Xanthomonadales; Xanthomonadaceae; Pseudoxanthomonas	UA
Bacteria; Proteobacteria; Gammaproteobacteria; Xanthomonadales; Rhodanobacteraceae; Rhodanobacter	UA
Bacteria; Firmicutes; Bacilli; Bacillales; Sporolactobacillaceae; Caenibacillus	UA
Bacteria; Firmicutes; Erysipelotrichia; Erysipelotrichales; Erysipelotrichaceae; Erysipelatoclostridium	SA
Bacteria; Firmicutes; Clostridia; Thermoanaerobacterales; Thermoanaerobacteraceae; Calderihabitans	SA
Bacteria; Firmicutes; Bacilli; Bacillales; Thermoactinomycetaceae; Desmospora	SA
Bacteria; Firmicutes; Clostridia; Eubacteriales; Oscillospiraceae; Harryflintia	SA
Bacteria; Balneolaeota; Balneolia; Balneolales; Balneolaceae; Rhodohalobacter	SA
Bacteria; Firmicutes; Bacilli; Lactobacillales; Enterococcaceae; Tetragenococcus	SA
Bacteria; Firmicutes; Bacilli; Bacillales; Unclassified Bacillales; Geomicrobium	SA
Bacteria; Firmicutes; Clostridia; Eubacteriales; Lachnospiraceae; Eisenbergiella	SA
Bacteria; Firmicutes; Bacilli; Bacillales; Paenibacillaceae; Xylanibacillus	SA
Bacteria; Proteobacteria; Gammaproteobacteria; Enterobacterales; Enterobacteriaceae; Atlantibacter	SA
Bacteria; Firmicutes; Bacilli; Bacillales; Bacillaceae; Weizmannia	SA
Bacteria; Actinobacteria; Coriobacteriia; Coriobacteriales; Atopobiaceae; Lancefieldella	SA
Bacteria; Firmicutes; Negativicutes; Selenomonadales; Sporomusaceae; Desulfosporomusa	SA
Bacteria; Firmicutes; Unclassified Firmicutes; Unclassified Firmicutes; Unclassified Firmicutes; Negativibacillus	SA
Bacteria; Actinobacteria; Coriobacteriia; Eggerthellales; Eggerthellaceae; Raoultibacter	SA
Bacteria; Firmicutes; Clostridia; Eubacteriales; Clostridiaceae; Oxobacter	SA
Bacteria; Proteobacteria; Gammaproteobacteria; Chromatiales; Ectothiorhodospiraceae; Natronocella	SA
Bacteria; Firmicutes; Clostridia; Eubacteriales; Peptococcaceae; Sporotomaculum	SA
Bacteria; Firmicutes; Bacilli; Bacillales; Bacillaceae; Falsibacillus	SA
Bacteria; Actinobacteria; Actinomycetia; Catenulisporales; Actinospicaceae; Actinocrinis	SA
Bacteria; Firmicutes; Clostridia; Eubacteriales; Eubacteriaceae; Alkalibacter	SA
Bacteria; Bacteroidetes; Bacteroidia; Bacteroidales; Porphyromonadaceae; Macellibacteroides	SA
Bacteria; Firmicutes; Clostridia; Eubacteriales; Clostridiaceae; Anoxynatronum	UAn
Bacteria; Actinobacteria; Actinomycetia; Micrococcales; Demequinaceae; Demequina	UAn
Bacteria; Planctomycetes; Planctomycetia; Isosphaerales; Isosphaeraceae; Isosphaera	UAn
Bacteria; Bacteroidetes; Bacteroidia; Bacteroidales; Prevotellaceae; Prevotellamassilia	UAn
Bacteria; Actinobacteria; Actinomycetia; Pseudonocardiales; Pseudonocardiaceae; Actinophytocola	UAn
Bacteria; Proteobacteria; Deltaproteobacteria; Desulfuromonadales; Geobacteraceae; Geomonas (Geobacteraceae)	UAn
Bacteria; Proteobacteria; Gammaproteobacteria; Xanthomonadales; Rhodanobacteraceae; Luteibacter	UAn
Bacteria; Proteobacteria; Deltaproteobacteria; Myxococcales; Polyangiaceae; Jahnella	UAn
Bacteria; Proteobacteria; Alphaproteobacteria; Hyphomicrobiales; Phyllobacteriaceae; Aquamicrobium	UAn
Bacteria; Proteobacteria; Alphaproteobacteria; Rhodospirillales; Rhodospirillaceae; Tistlia	UAn
Bacteria; Proteobacteria; Betaproteobacteria; Burkholderiales; Comamonadaceae; Acidovorax	UAn
Bacteria; Actinobacteria; Actinomycetia; Streptosporangiales; Streptosporangiaceae; Sinosporangium	UAn
Bacteria; Actinobacteria; Actinomycetia; Sporichthyales; Sporichthyaceae; Sporichthya	UAn
Bacteria; Bacteroidetes; Sphingobacteriia; Sphingobacteriales; Sphingobacteriaceae; Daejeonella	UAn
Bacteria; Proteobacteria; Alphaproteobacteria; Hyphomicrobiales; Bradyrhizobiaceae; Rhodopseudomonas	UAn
Bacteria; Proteobacteria; Betaproteobacteria; Burkholderiales; Burkholderiaceae; Burkholderia	UAn
Bacteria; Spirochaetes; Spirochaetia; Spirochaetales; Spirochaetaceae; Treponema	UAn
Bacteria; Firmicutes; Clostridia; Eubacteriales; Peptostreptococcaceae; Asaccharospora	UAn
Bacteria; Bacteroidetes; Bacteroidia; Marinilabiliales; Marinilabiliaceae; Geofilum	UAn
Bacteria; Proteobacteria; Alphaproteobacteria; Rhodospirillales; Acetobacteraceae; Caldovatus	UAn
Bacteria; Proteobacteria; Alphaproteobacteria; Sphingomonadales; Sphingomonadaceae; Blastomonas	UAn
Bacteria; Proteobacteria; Alphaproteobacteria; Hyphomicrobiales; Phyllobacteriaceae; Nitratireductor	UAn
Bacteria; Bacteroidetes; Sphingobacteriia; Sphingobacteriales; Sphingobacteriaceae; Pedobacter	UAn
Bacteria; Proteobacteria; Gammaproteobacteria; Xanthomonadales; Rhodanobacteraceae; Fulvimonas	UAn
Bacteria; Actinobacteria; Actinomycetia; Micromonosporales; Micromonosporaceae; Couchioplanes	UAn
Bacteria; Firmicutes; Bacilli; Bacillales; Planococcaceae; Metasolibacillus	UAn
Bacteria; Bacteroidetes; Sphingobacteriia; Sphingobacteriales; Sphingobacteriaceae; Mucilaginibacter	UAn
Bacteria; Actinobacteria; Actinomycetia; Micrococcales; Micrococcaceae; Tersicoccus	UAn

Bacteria; Proteobacteria; Alphaproteobacteria; Rhodobacterales; Rhodobacteraceae; Rhodobacter	UAn
Bacteria; Firmicutes; Bacilli; Bacillales; Bacillaceae; Hydrogenibacillus	UAn
Bacteria; Chloroflexi; Thermomicrobia; Sphaerobacterales; Sphaerobacteraceae; Nitrolancea	UAn
Bacteria; Armatimonadetes; Armatimonadia; Armatimonadales; Armatimonadaceae; Armatimonas	UAn
Bacteria; Firmicutes; Clostridia; Eubacteriales; Lachnospiraceae; Abyssivirga	UAn
Bacteria; Proteobacteria; Gammaproteobacteria; Chromatiales; Ectothiorhodospiraceae; Inmirania	UAn
Bacteria; Firmicutes; Clostridia; Eubacteriales; Peptostreptococcaceae; Peptoclostridium	UAn
Bacteria; Proteobacteria; Alphaproteobacteria; Rhodospirillales; Rhodospirillaceae; Taonella	UAn
Bacteria; Bacteroidetes; Flavobacteriia; Flavobacteriales; Weeksellaceae; Kaistella	UAn
Bacteria; Proteobacteria; Alphaproteobacteria; Sphingomonadales; Erythrobacteraceae; Aurantiacibacter	UAn
Bacteria; Firmicutes; Bacilli; Bacillales; Thermoactinomycetaceae; Polycladomyces	UAn
Bacteria; Actinobacteria; Actinomycetia; Streptomycetales; Streptomycetaceae; Embleya	UAn
Bacteria; Firmicutes; Negativicutes; Selenomonadales; Sporomusaceae; Pelosinus	UAn
Bacteria; Proteobacteria; Alphaproteobacteria; Hyphomicrobiales; Rhizobiaceae; Ensifer	UAn
Bacteria; Acidobacteria; Acidobacteriia; Acidobacteriales; Acidobacteriaceae; Acidicapsa	UAn
Bacteria; Bacteroidetes; Cytophagia; Cytophagales; Cytophagaceae; Sporocytophaga	UAn
Bacteria; Actinobacteria; Actinomycetia; Corynebacteriales; Mycobacteriaceae; Mycolicibacter	UAn
Bacteria; Proteobacteria; Gammaproteobacteria; Pasteurellales; Pasteurellaceae; Glaesserella	UAn
Bacteria; Firmicutes; Bacilli; Bacillales; Bacillaceae; Aeribacillus	UAn
Bacteria; Bacteroidetes; Unclassified Bacteroidetes; Bacteroidetes Order II. Incertae sedis; Rhodothermaceae; Rhodothermus	UAn
Bacteria; Actinobacteria; Actinomycetia; Pseudonocardiales; Pseudonocardiaceae; Kutzneria	UAn
Bacteria; Proteobacteria; Alphaproteobacteria; Rhodobacterales; Rhodobacteraceae; Xinfangfangia	UAn
Bacteria; Chloroflexi; Unclassified Chloroflexi; Unclassified Chloroflexi; Unclassified Chloroflexi; Thermobaculum	UAn
Bacteria; Actinobacteria; Actinomycetia; Propionibacteriales; Nocardioidaceae; Aeromicrobium	UAn
Bacteria; Proteobacteria; Gammaproteobacteria; Enterobacterales; Enterobacteriaceae; Raoultella	UAn
Bacteria; Firmicutes; Clostridia; Eubacteriales; Clostridiaceae; Hungatella	UAn
Bacteria; Proteobacteria; Deltaproteobacteria; Desulfobacterales; Desulfobacteraceae; Desulfonema	UAn
Bacteria; Planctomycetes; Planctomycetia; Pirellulales; Pirellulaceae; Roseimaritima	UAn
Bacteria; Proteobacteria; Deltaproteobacteria; Myxococcales; Archangiaceae; Hyalangium	UAn
Bacteria; Balneolaeota; Balneolia; Balneolales; Balneolaceae; Aliifodinibius	UAn
Bacteria; Proteobacteria; Gammaproteobacteria; Enterobacterales; Enterobacteriaceae; Shigella	UAn
Bacteria; Proteobacteria; Gammaproteobacteria; Xanthomonadales; Rhodanobacteraceae; Dyella	UAn
Bacteria; Proteobacteria; Deltaproteobacteria; Desulfobacterales; Desulfobacteraceae; Desulfococcus	UAn
Bacteria; Actinobacteria; Actinomycetia; Micrococcales; Microbacteriaceae; Agromyces	UAn
Bacteria; Proteobacteria; Gammaproteobacteria; Acidiferrobacterales; Acidiferrobacteraceae; Sulfuricaulis	UAn
Bacteria; Proteobacteria; Deltaproteobacteria; Unclassified Deltaproteobacteria; Unclassified Deltaproteobacteria; Deferrisoma	UAn
Bacteria; Acidobacteria; Acidobacteriia; Acidobacteriales; Acidobacteriaceae; Silvibacterium	UAn
Bacteria; Firmicutes; Clostridia; Eubacteriales; Clostridiales Family XIII. Incertae Sedis; Aminipila	UAn
Bacteria; Proteobacteria; Gammaproteobacteria; Xanthomonadales; Rhodanobacteraceae; Mizugakiibacter	UAn
Bacteria; Bacteroidetes; Cytophagia; Cytophagales; Cytophagaceae; Ravibacter	UAn
Bacteria; Actinobacteria; Actinomycetia; Streptosporangiales; Streptosporangiaceae; Thermopolyspora	UAn
Bacteria; Verrucomicrobia; Unclassified Verrucomicrobia; Unclassified Verrucomicrobia; Unclassified Verrucomicrobia; Methylacidimicrobium	UAn
Bacteria; Proteobacteria; Gammaproteobacteria; Enterobacterales; Erwiniaceae; Erwinia	UAn
Bacteria; Firmicutes; Bacilli; Bacillales; Bacillaceae; Saliterribacillus	UAn
Bacteria; Firmicutes; Bacilli; Bacillales; Bacillaceae; Salipaludibacillus	UAn
Bacteria; Proteobacteria; Betaproteobacteria; Burkholderiales; Oxalobacteraceae; Oxalicibacterium	UAn
Bacteria; Proteobacteria; Gammaproteobacteria; Xanthomonadales; Xanthomonadaceae; Arenimonas	UAn
Bacteria; Actinobacteria; Actinomycetia; Micrococcales; Promicromonosporaceae; Promicromonospora	UAn
Bacteria; Actinobacteria; Actinomycetia; Micrococcales; Promicromonosporaceae; Isoptericola	UAn
Bacteria; Proteobacteria; Betaproteobacteria; Burkholderiales; Oxalobacteraceae; Undibacterium	UAn
Bacteria; Bacteroidetes; Bacteroidia; Marinilabiliales; Marinilabiliaceae; Mangroviflexus	UAn
Bacteria; Firmicutes; Negativicutes; Selenomonadales; Sporomusaceae; Anaerosinus	UAn
Bacteria; Proteobacteria; Deltaproteobacteria; Myxococcales; Polyangiaceae; Aetherobacter	UAn
Bacteria; Bacteroidetes; Sphingobacteriia; Sphingobacteriales; Sphingobacteriaceae; Solitalea	UAn
Bacteria; Firmicutes; Clostridia; Eubacteriales; Peptostreptococcaceae; Acetoanaerobium	UAn
Bacteria; Firmicutes; Clostridia; Halanaerobiales; Halanaerobiaceae; Halothermothrix	UAn
Bacteria; Proteobacteria; Betaproteobacteria; Burkholderiales; Oxalobacteraceae; Duganella	UAn
Bacteria; Chloroflexi; Anaerolineae; Anaerolineales; Anaerolineaceae; Ornatilinea	UAn
Bacteria; Bacteroidetes; Cytophagia; Cytophagales; Cytophagaceae; Rhodocytophaga	UAn
Bacteria; Proteobacteria; Gammaproteobacteria; Xanthomonadales; Rhodanobacteraceae; Frateuria	UAn
Bacteria; Proteobacteria; Alphaproteobacteria; Rhodospirillales; Rhodospirillaceae; Elstera	UAn
Bacteria; Proteobacteria; Alphaproteobacteria; Sphingomonadales; Sphingosinicellaceae; Sphingosinicella	UAn
Bacteria; Proteobacteria; Alphaproteobacteria; Rhodospirillales; Rhodospirillaceae; Inquilinus	UAn
Bacteria; Actinobacteria; Actinomycetia; Micromonosporales; Micromonosporaceae; Catellatospora	UAn
Bacteria; Firmicutes; Clostridia; Eubacteriales; Oscillospiraceae; Subdoligranulum	SAn
Bacteria; Firmicutes; Bacilli; Lactobacillales; Lactobacillaceae; Lapidilactobacillus	SAn
Bacteria; Firmicutes; Clostridia; Eubacteriales; Eubacteriaceae; Anaerofustis	SAn
Bacteria; Actinobacteria; Actinomycetia; Actinomycetales; Actinomycetaceae; Actinomyces	SAn
Bacteria; Firmicutes; Clostridia; Eubacteriales; Lachnospiraceae; Lachnobacterium	SAn
Bacteria; Firmicutes; Tissierellia; Tissierellales; Gottschalkiaceae; Gottschalkia	SAn
Bacteria; Proteobacteria; Alphaproteobacteria; Hyphomicrobiales; Chelatococcaceae; Chelatococcus	SAn
Bacteria; Proteobacteria; Betaproteobacteria; Burkholderiales; Comamonadaceae; Pelomonas	SAn
Bacteria; Fusobacteria; Fusobacteriia; Fusobacteriales; Fusobacteriaceae; Fusobacterium	SAn
Bacteria; Proteobacteria; Deltaproteobacteria; Desulfovibrionales; Desulfovibrionaceae; Mailhella	SAn
Bacteria; Firmicutes; Clostridia; Eubacteriales; Peptococcaceae; Dehalobacter	SAn
Bacteria; Proteobacteria; Alphaproteobacteria; Hyphomicrobiales; Methylobacteriaceae; Methylorubrum	SAn
Bacteria; Actinobacteria; Actinomycetia; Micrococcales; Micrococcaceae; Falsarthrobacter	SAn

Bacteria; Firmicutes; Clostridia; Eubacteriales; Oscillospiraceae; Anaerofilum	SAn
Bacteria; Firmicutes; Bacilli; Bacillales; Bacillaceae; Calditerricola	SAn
Bacteria; Firmicutes; Clostridia; Eubacteriales; Peptococcaceae; Desulfocucumis	SAn
Bacteria; Firmicutes; Erysipelotrichia; Erysipelotrichales; Erysipelotrichaceae; Faecalicoccus	SAn
Bacteria; Firmicutes; Bacilli; Bacillales; Bacillaceae; Piscibacillus	SAn
Bacteria; Actinobacteria; Actinomycetia; Corynebacteriales; Dietziaceae; Dietzia	SAn
Bacteria; Firmicutes; Bacilli; Lactobacillales; Carnobacteriaceae; Granulicatella	SAn
Bacteria; Proteobacteria; Alphaproteobacteria; Hyphomicrobiales; Beijerinckiaceae; Methylocapsa	SAn
Bacteria; Proteobacteria; Alphaproteobacteria; Hyphomicrobiales; Bradyrhizobiaceae; Afipia	SAn
Bacteria; Firmicutes; Bacilli; Bacillales; Bacillaceae; Amphibacillus	SAn
Bacteria; Firmicutes; Bacilli; Bacillales; Bacillaceae; Terribacillus	SAn
Bacteria; Firmicutes; Clostridia; Thermoanaerobacterales; Thermoanaerobacteraceae; Thermodesulfitimonas	SAn
Bacteria; Firmicutes; Bacilli; Bacillales; Sporolactobacillaceae; Sporolactobacillus	SAn
Bacteria; Proteobacteria; Gammaproteobacteria; Xanthomonadales; Xanthomonadaceae; Xanthomonas	SAn
