# Supplementary material for: Composition and functional diversity of bacterial communities during swine carcass decomposition
Source: Anim Biosci. 2023 Jun 26;36(9):1453–64. doi: 10.5713/ab.23.0140 (PMC10472150; doi:10.5713/ab.23.0140)
Supplement: Supplementary file 3 [file ab-23-0140-Supplementary-Table-2.pdf]

Table S2. Taxonomic classification of shared bacterial genera present between UA, SA, UAn, and SAn samples

Taxonomy	Burial set-up
Bacteria; Bacteroidetes; Bacteroidia; Bacteroidales; Rikenellaceae; Alistipes	UA|SA
Bacteria; Firmicutes; Clostridia; Eubacteriales; Unclassified Eubacteriales; Natranaerovirga	UA|SA
Bacteria; Proteobacteria; Gammaproteobacteria; Pseudomonadales; Moraxellaceae; Acinetobacter	UA|SA
Bacteria; Proteobacteria; Betaproteobacteria; Burkholderiales; Burkholderiaceae; Ralstonia	UA|SA
Bacteria; Proteobacteria; Betaproteobacteria; Burkholderiales; Alcaligenaceae; Achromobacter	UA|SA
Bacteria; Proteobacteria; Alphaproteobacteria; Sphingomonadales; Sphingomonadaceae; Novosphingobium	UA|SA
Bacteria; Proteobacteria; Betaproteobacteria; Burkholderiales; Comamonadaceae; Comamonas	UA|SA
Bacteria; Firmicutes; Bacilli; Bacillales; Thermoactinomycetaceae; Geothermomicrobium	UAn|SAn
Bacteria; Proteobacteria; Betaproteobacteria; Burkholderiales; Comamonadaceae; Ramlibacter	UAn|SAn
Bacteria; Firmicutes; Bacilli; Bacillales; Planococcaceae; Jeotgalibacillus	UAn|SAn
Bacteria; Firmicutes; Negativicutes; Veillonellales; Veillonellaceae; Allisonella	UAn|SAn
Bacteria; Actinobacteria; Actinomycetia; Micrococcales; Micrococcaceae; Rothia	UAn|SAn
Bacteria; Firmicutes; Bacilli; Bacillales; Paenibacillaceae; Thermobacillus	UAn|SAn
Bacteria; Firmicutes; Clostridia; Eubacteriales; Unclassified Eubacteriales; Neglecta	UAn|SAn
Bacteria; Firmicutes; Clostridia; Eubacteriales; Oscillospiraceae; Phocea	SA|SAn
Bacteria; Firmicutes; Clostridia; Eubacteriales; Peptococcaceae; Desulfotomaculum	SA|SAn
Bacteria; Firmicutes; Bacilli; Lactobacillales; Lactobacillaceae; Lacticaseibacillus	SA|SAn
Bacteria; Firmicutes; Bacilli; Bacillales; Bacillaceae; Aquibacillus	SA|SAn
Bacteria; Actinobacteria; Coriobacteriia; Eggerthellales; Eggerthellaceae; Slackia	SA|SAn
Bacteria; Firmicutes; Clostridia; Eubacteriales; Peptococcaceae; Desulfosporosinus	SA|SAn
Bacteria; Firmicutes; Bacilli; Bacillales; Paenibacillaceae; Fontibacillus	SA|SAn
Bacteria; Actinobacteria; Actinomycetia; Propionibacteriales; Propionibacteriaceae; Propionibacterium	SA|SAn
Bacteria; Firmicutes; Tissierellia; Tissierellales; Peptoniphilaceae; Anaerococcus	SA|SAn
Bacteria; Firmicutes; Bacilli; Bacillales; Bacillaceae; Anaerobacillus	SA|SAn
Bacteria; Proteobacteria; Betaproteobacteria; Burkholderiales; Oxalobacteraceae; Pseudoduganella	UA|UAn
Bacteria; Proteobacteria; Betaproteobacteria; Burkholderiales; Unclassified Burkholderiales; Tepidimonas	UA|UAn
Bacteria; Proteobacteria; Gammaproteobacteria; Chromatiales; Granulosicoccaceae; Sulfuriflexus	UA|UAn
Bacteria; Proteobacteria; Betaproteobacteria; Burkholderiales; Unclassified Burkholderiales; Ideonella	UA|UAn
Bacteria; Actinobacteria; Actinomycetia; Micromonosporales; Micromonosporaceae; Virgisporangium	UA|UAn
Bacteria; Proteobacteria; Alphaproteobacteria; Hyphomicrobiales; Stappiaceae; Labrenzia	UA|UAn
Bacteria; Proteobacteria; Alphaproteobacteria; Hyphomicrobiales; Xanthobacteraceae; Labrys	UA|UAn
Bacteria; Proteobacteria; Deltaproteobacteria; Myxococcales; Archangiaceae; Vitiosangium	UA|UAn
Bacteria; Cyanobacteria; Unclassified Cyanobacteria; Pleurocapsales; Dermocarpellaceae; Stanieria	UA|UAn
Bacteria; Proteobacteria; Alphaproteobacteria; Hyphomicrobiales; Parvibaculaceae; Rhodoligotrophos	UA|UAn
Bacteria; Firmicutes; Clostridia; Eubacteriales; Clostridiaceae; Abyssisolibacter	UA|UAn
Bacteria; Proteobacteria; Betaproteobacteria; Burkholderiales; Comamonadaceae; Caldimonas	UA|UAn
Bacteria; Actinobacteria; Actinomycetia; Catenulisporales; Catenulisporaceae; Catenulispora	UA|UAn
Bacteria; Proteobacteria; Alphaproteobacteria; Holosporales; Caedimonadaceae; Caedimonas	UA|UAn
Bacteria; Proteobacteria; Alphaproteobacteria; Hyphomicrobiales; Hyphomicrobiaceae; Pedomicrobium	UA|UAn
Bacteria; Bacteroidetes; Chitinophagia; Chitinophagales; Chitinophagaceae; Sediminibacterium	UA|UAn
Bacteria; Proteobacteria; Gammaproteobacteria; Alteromonadales; Alteromonadaceae; Marinobacter	UA|UAn
Bacteria; Proteobacteria; Alphaproteobacteria; Hyphomicrobiales; Rhizobiaceae; Agrobacterium	UA|UAn
Bacteria; Acidobacteria; Acidobacteriia; Bryobacterales; Bryobacteraceae; Bryobacter	UA|UAn
Bacteria; Firmicutes; Bacilli; Bacillales; Thermoactinomycetaceae; Risungbinella	UA|UAn
Bacteria; Chlamydiae; Chlamydiia; Parachlamydiales; Simkaniaceae; Simkania	UA|UAn
Bacteria; Proteobacteria; Alphaproteobacteria; Rhodobacterales; Rhodobacteraceae; Oceanicella	UA|UAn
Bacteria; Proteobacteria; Alphaproteobacteria; Rhodospirillales; Rhodospirillaceae; Aliidongia	UA|UAn
Bacteria; Chloroflexi; Caldilineae; Caldilineales; Caldilineaceae; Litorilinea	UA|UAn
Bacteria; Actinobacteria; Actinomycetia; Pseudonocardiales; Pseudonocardiaceae; Actinomycetospora	UA|UAn
Bacteria; Actinobacteria; Actinomycetia; Micromonosporales; Micromonosporaceae; Phytohabitans	UA|UAn
Bacteria; Proteobacteria; Alphaproteobacteria; Rhodospirillales; Acetobacteraceae; Acidibrevibacterium	UA|UAn
Bacteria; Bacteroidetes; Cytophagia; Cytophagales; Hymenobacteraceae; Adhaeribacter	UA|UAn
Bacteria; Proteobacteria; Gammaproteobacteria; Enterobacterales; Enterobacteriaceae; Enterobacter	UA|UAn
Bacteria; Proteobacteria; Deltaproteobacteria; Myxococcales; Nannocystaceae; Nannocystis	UA|UAn
Bacteria; Actinobacteria; Actinomycetia; Micrococcales; Intrasporangiaceae; Terrabacter	UA|UAn
Bacteria; Chlamydiae; Chlamydiia; Parachlamydiales; Parachlamydiaceae; Neochlamydia	UA|UAn
Bacteria; Actinobacteria; Actinomycetia; Micromonosporales; Micromonosporaceae; Asanoa	UA|UAn
Bacteria; Actinobacteria; Actinomycetia; Micromonosporales; Micromonosporaceae; Actinocatenispora	UA|UAn
Bacteria; Actinobacteria; Thermoleophilia; Solirubrobacterales; Conexibacteraceae; Conexibacter	UA|UAn
Bacteria; Actinobacteria; Actinomycetia; Micrococcales; Promicromonosporaceae; Xylanibacterium	UA|UAn
Bacteria; Proteobacteria; Alphaproteobacteria; Sphingomonadales; Sphingomonadaceae; Sphingopyxis	UA|UAn
Bacteria; Proteobacteria; Betaproteobacteria; Nitrosomonadales; Nitrosomonadaceae; Nitrosomonas	UA|UAn
Bacteria; Actinobacteria; Actinomycetia; Geodermatophilales; Geodermatophilaceae; Modestobacter	UA|UAn
Bacteria; Firmicutes; Clostridia; Eubacteriales; Clostridiaceae; Falcatimonas	UA|UAn
Bacteria; Actinobacteria; Actinomycetia; Acidothermales; Acidothermaceae; Acidothermus	UA|UAn
Bacteria; Proteobacteria; Deltaproteobacteria; Myxococcales; Vulgatibacteraceae; Vulgatibacter	UA|UAn
Bacteria; Actinobacteria; Actinomycetia; Jatrophihabitantales; Jatrophihabitantaceae; Jatrophihabitans	UA|UAn
Bacteria; Proteobacteria; Gammaproteobacteria; Xanthomonadales; Xanthomonadaceae; Chujaibacter	UA|UAn
Bacteria; Actinobacteria; Actinomycetia; Corynebacteriales; Nocardiaceae; Nocardia	UA|UAn
Bacteria; Proteobacteria; Deltaproteobacteria; Myxococcales; Polyangiaceae; Byssovorax	UA|UAn
Bacteria; Proteobacteria; Betaproteobacteria; Burkholderiales; Oxalobacteraceae; Herbaspirillum	UA|UAn
Bacteria; Firmicutes; Negativicutes; Selenomonadales; Sporomusaceae; Sporomusa	UA|UAn
Bacteria; Proteobacteria; Alphaproteobacteria; Hyphomicrobiales; Methylocystaceae; Methylocystis	UA|UAn
Bacteria; Proteobacteria; Betaproteobacteria; Burkholderiales; Unclassified Burkholderiales; Rubrivivax	UA|UAn
Bacteria; Proteobacteria; Alphaproteobacteria; Rhodospirillales; Azospirillaceae; Azospirillum	UA|UAn
Bacteria; Actinobacteria; Actinomycetia; Streptosporangiales; Streptosporangiaceae; Streptosporangium	UA|UAn
Bacteria; Proteobacteria; Gammaproteobacteria; Nevskiales; Steroidobacteraceae; Povalibacter	UA|UAn
Bacteria; Proteobacteria; Deltaproteobacteria; Desulfuromonadales; Geobacteraceae; Geoalkalibacter	UA|UAn
Bacteria; Proteobacteria; Deltaproteobacteria; Myxococcales; Anaeromyxobacteraceae; Anaeromyxobacter	UA|UAn
Bacteria; Proteobacteria; Deltaproteobacteria; Desulfuromonadales; Geobacteraceae; Geobacter	UA|UAn
Bacteria; Chlamydiae; Chlamydiia; Parachlamydiales; Parachlamydiaceae; Candidatus Protochlamydia	UA|UAn
Bacteria; Actinobacteria; Thermoleophilia; Thermoleophilales; Thermoleophilaceae; Thermoleophilum	UA|UAn
Bacteria; Proteobacteria; Gammaproteobacteria; Cellvibrionales; Microbulbiferaceae; Microbulbifer	UA|UAn
Bacteria; Actinobacteria; Actinomycetia; Micrococcales; Micrococcaceae; Arthrobacter	UA|UAn
Bacteria; Proteobacteria; Alphaproteobacteria; Caulobacterales; Caulobacteraceae; Phenylobacterium	UA|UAn
Bacteria; Proteobacteria; Alphaproteobacteria; Rhodospirillales; Acetobacteraceae; Stella	UA|UAn
Bacteria; Thermodesulfobacteria; Thermodesulfobacteria; Thermodesulfobacteriales; Thermodesulfobacteriaceae; Thermodesulfatator	UA|UAn
Bacteria; Planctomycetes; Planctomycetia; Isosphaerales; Isosphaeraceae; Singulisphaera	UA|UAn
Bacteria; Proteobacteria; Alphaproteobacteria; Rhodospirillales; Rhodospirillaceae; Magnetospirillum	UA|UAn
Bacteria; Proteobacteria; Deltaproteobacteria; Myxococcales; Polyangiaceae; Chondromyces	UA|UAn
Bacteria; Chloroflexi; Chloroflexia; Chloroflexales; Oscillochloridaceae; Oscillochloris	UA|UAn
Bacteria; Chloroflexi; Ktedonobacteria; Thermogemmatisporales; Thermogemmatisporaceae; Thermogemmatispora	UA|UAn
Bacteria; Proteobacteria; Alphaproteobacteria; Rhodospirillales; Azospirillaceae; Skermanella	UA|UAn
Bacteria; Proteobacteria; Alphaproteobacteria; Rhodospirillales; Rhodospirillaceae; Oceanibaculum	UA|UAn
Bacteria; Proteobacteria; Alphaproteobacteria; Hyphomicrobiales; Hyphomicrobiaceae; Rhodomicrobium	UA|UAn
Bacteria; Dictyoglomi; Dictyoglomia; Dictyoglomales; Dictyoglomaceae; Dictyoglomus	UA|UAn
Bacteria; Actinobacteria; Actinomycetia; Streptosporangiales; Thermomonosporaceae; Thermostaphylospora	UA|UAn
Bacteria; Proteobacteria; Alphaproteobacteria; Sphingomonadales; Erythrobacteraceae; Erythrobacter	UA|UAn
Bacteria; Proteobacteria; Alphaproteobacteria; Hyphomicrobiales; Hyphomicrobiaceae; Methyloceanibacter	UA|UAn
Bacteria; Proteobacteria; Betaproteobacteria; Rhodocyclales; Rhodocyclaceae; Oryzomicrobium	UA|UAn
Bacteria; Verrucomicrobia; Terrimicrobia; Terrimicrobiales; Terrimicrobiaceae; Terrimicrobium	UA|UAn
Bacteria; Proteobacteria; Alphaproteobacteria; Hyphomicrobiales; Kaistiaceae; Bauldia	UA|UAn
Bacteria; Candidatus Melainabacteria; Unclassified Candidatus Melainabacteria; Vampirovibrionales; Unclassified Vampirovibrionales; Vampirovibrio	UA|UAn
Bacteria; Proteobacteria; Alphaproteobacteria; Micropepsales; Micropepsaceae; Rhizomicrobium	UA|UAn
Bacteria; Actinobacteria; Thermoleophilia; Solirubrobacterales; Parviterribacteraceae; Parviterribacter	UA|UAn
Bacteria; Acidobacteria; Blastocatellia; Blastocatellales; Pyrinomonadaceae; Pyrinomonas	UA|UAn
Bacteria; Chlamydiae; Chlamydiia; Parachlamydiales; Waddliaceae; Waddlia	UA|UAn
Bacteria; Bacteroidetes; Flavobacteriia; Flavobacteriales; Flavobacteriaceae; Flavobacterium	UA|UAn
Bacteria; Actinobacteria; Actinomycetia; Pseudonocardiales; Pseudonocardiaceae; Amycolatopsis	UA|UAn
Bacteria; Proteobacteria; Deltaproteobacteria; Myxococcales; Unclassified Myxococcales; Minicystis	UA|UAn
Bacteria; Firmicutes; Bacilli; Bacillales; Thermoactinomycetaceae; Lihuaxuella	UA|UAn
Bacteria; Actinobacteria; Actinomycetia; Micrococcales; Intrasporangiaceae; Oryzihumus	UA|UAn
Bacteria; Actinobacteria; Actinomycetia; Micrococcales; Microbacteriaceae; Cryobacterium	UA|UAn
Bacteria; Proteobacteria; Alphaproteobacteria; Hyphomicrobiales; Boseaceae; Bosea	UA|UAn

Bacteria; Actinobacteria; Actinomycetia; Pseudonocardiales; Pseudonocardiaceae; Saccharopolyspora	UA|UAn
Bacteria; Proteobacteria; Betaproteobacteria; Burkholderiales; Burkholderiaceae; Trinickia	UA|UAn
Bacteria; Actinobacteria; Actinomycetia; Micromonosporales; Micromonosporaceae; Hamadaea	UA|UAn
Bacteria; Acidobacteria; Blastocatellia; Blastocatellales; Pyrinomonadaceae; Brevitalea	UA|UAn
Bacteria; Actinobacteria; Actinomycetia; Streptosporangiales; Streptosporangiaceae; Thermobispora	UA|UAn
Bacteria; Planctomycetes; Planctomycetia; Planctomycetales; Planctomycetaceae; Rubinisphaera	UA|UAn
Bacteria; Actinobacteria; Actinomycetia; Pseudonocardiales; Pseudonocardiaceae; Herbihabitans	UA|UAn
Bacteria; Bacteroidetes; Chitinophagia; Chitinophagales; Chitinophagaceae; Niastella	UA|UAn
Bacteria; Proteobacteria; Alphaproteobacteria; Hyphomicrobiales; Beijerinckiaceae; Methylovirgula	UA|UAn
Bacteria; Actinobacteria; Actinomycetia; Corynebacteriales; Mycobacteriaceae; Mycobacterium	UA|UAn
Bacteria; Proteobacteria; Alphaproteobacteria; Hyphomicrobiales; Rhizobiaceae; Rhizobium	UA|UAn
Bacteria; Actinobacteria; Nitriliruptoria; Euzebyales; Euzebyaceae; Euzebya	UA|UAn
Bacteria; Proteobacteria; Gammaproteobacteria; Xanthomonadales; Rhodanobacteraceae; Rudaea	UA|UAn
Bacteria; Planctomycetes; Phycisphaerae; Tepidisphaerales; Tepidisphaeraceae; Tepidisphaera	UA|UAn
Bacteria; Firmicutes; Clostridia; Eubacteriales; Lachnospiraceae; Herbinix	UA|UAn
Bacteria; Planctomycetes; Planctomycetia; Isosphaerales; Isosphaeraceae; Tundrisphaera	UA|UAn
Bacteria; Chloroflexi; Chloroflexia; Chloroflexales; Roseiflexaceae; Roseiflexus	UA|UAn
Bacteria; Verrucomicrobia; Verrucomicrobiae; Verrucomicrobiales; Verrucomicrobia subdivision 3; Limisphaera	UA|UAn
Bacteria; Proteobacteria; Alphaproteobacteria; Hyphomicrobiales; Methylocystaceae; Methylosinus	UA|UAn
Bacteria; Proteobacteria; Gammaproteobacteria; Chromatiales; Unclassified Chromatiales; Thiohalobacter	UA|UAn
Bacteria; Proteobacteria; Betaproteobacteria; Burkholderiales; Burkholderiaceae; Caballeronia	UA|UAn
Bacteria; Proteobacteria; Alphaproteobacteria; Hyphomicrobiales; Unclassified Hyphomicrobiales; Nordella	UA|UAn
Bacteria; Proteobacteria; Alphaproteobacteria; Rhodospirillales; Rhodospirillaceae; Ferruginivarius	UA|UAn
Bacteria; Bacteroidetes; Chitinophagia; Chitinophagales; Chitinophagaceae; Parafilimonas	UA|UAn
Bacteria; Planctomycetes; Planctomycetia; Planctomycetales; Planctomycetaceae; Gimesia	UA|UAn
Bacteria; Proteobacteria; Betaproteobacteria; Nitrosomonadales; Sterolibacteriaceae; Methyloversatilis	UA|UAn
Bacteria; Bacteroidetes; Bacteroidia; Bacteroidales; Prevotellaceae; Paraprevotella	UA|UAn
Bacteria; Proteobacteria; Betaproteobacteria; Burkholderiales; Burkholderiaceae; Paraburkholderia	UA|UAn
Bacteria; Gemmatimonadetes; Gemmatimonadetes; Gemmatimonadales; Gemmatimonadaceae; Gemmatirosa	UA|UAn
Bacteria; Proteobacteria; Alphaproteobacteria; Rhodospirillales; Rhodospirillaceae; Lacibacterium	UA|UAn
Bacteria; Actinobacteria; Actinomycetia; Corynebacteriales; Nocardiaceae; Rhodococcus	UA|UAn
Bacteria; Planctomycetes; Planctomycetia; Pirellulales; Thermoguttaceae; Thermogutta	UA|UAn
Bacteria; Firmicutes; Clostridia; Eubacteriales; Peptococcaceae; Desulfofarcimen	UA|UAn
Bacteria; Chloroflexi; Chloroflexia; Chloroflexales; Chloroflexaceae; Chloroflexus	UA|UAn
Bacteria; Actinobacteria; Actinomycetia; Streptosporangiales; Nocardiopsaceae; Thermobifida	UA|UAn
Bacteria; Proteobacteria; Deltaproteobacteria; Syntrophobacterales; Syntrophaceae; Desulfomonile	UA|UAn
Bacteria; Proteobacteria; Deltaproteobacteria; Myxococcales; Kofleriaceae; Haliangium	UA|UAn
Bacteria; Synergistetes; Synergistia; Synergistales; Synergistaceae; Thermanaerovibrio	UA|UAn
Bacteria; Chloroflexi; Anaerolineae; Anaerolineales; Anaerolineaceae; Bellilinea	UA|UAn
Bacteria; Proteobacteria; Alphaproteobacteria; Hyphomicrobiales; Roseiarcaceae; Roseiarcus	UA|UAn
Bacteria; Armatimonadetes; Chthonomonadetes; Chthonomonadales; Chthonomonadaceae; Chthonomonas	UA|UAn
Bacteria; Proteobacteria; Gammaproteobacteria; Legionellales; Coxiellaceae; Aquicella	UA|UAn
Bacteria; Proteobacteria; Alphaproteobacteria; Rhodospirillales; Unclassified Rhodospirillales; Reyranella	UA|UAn
Bacteria; Proteobacteria; Deltaproteobacteria; Myxococcales; Polyangiaceae; Sorangium	UA|UAn
Bacteria; Planctomycetes; Planctomycetia; Gemmatales; Gemmataceae; Zavarzinella	UA|UAn
Bacteria; Firmicutes; Clostridia; Eubacteriales; Proteinivoraceae; Proteinivorax	UA|UAn
Bacteria; Proteobacteria; Alphaproteobacteria; Rhodospirillales; Geminicoccaceae; Arboricoccus	UA|UAn
Bacteria; Proteobacteria; Alphaproteobacteria; Rhodospirillales; Rhodospirillaceae; Fodinicurvata	UA|UAn
Bacteria; Actinobacteria; Acidimicrobiia; Acidimicrobiales; Ilumatobacteraceae; Ilumatobacter	UA|UAn
Bacteria; Proteobacteria; Betaproteobacteria; Rhodocyclales; Azonexaceae; Ferribacterium	UA|UAn
Bacteria; Proteobacteria; Gammaproteobacteria; Xanthomonadales; Xanthomonadaceae; Lysobacter	UA|UAn
Bacteria; Proteobacteria; Alphaproteobacteria; Hyphomicrobiales; Rhodobiaceae; Tepidamorphus	UA|UAn
Bacteria; Proteobacteria; Betaproteobacteria; Rhodocyclales; Zoogloeaceae; Azoarcus	UA|UAn
Bacteria; Proteobacteria; Alphaproteobacteria; Hyphomicrobiales; Alsobacteraceae; Alsobacter	UA|UAn
Bacteria; Planctomycetes; Planctomycetia; Pirellulales; Pirellulaceae; Pirellula	UA|UAn
Bacteria; Proteobacteria; Alphaproteobacteria; Rhodospirillales; Azospirillaceae; Desertibacter	UA|UAn
Bacteria; Actinobacteria; Actinomycetia; Micrococcales; Intrasporangiaceae; Intrasporangium	UA|UAn
Bacteria; Proteobacteria; Alphaproteobacteria; Hyphomicrobiales; Rhizobiaceae; Shinella	UA|UAn
Bacteria; Actinobacteria; Actinomycetia; Micrococcales; Cellulomonadaceae; Cellulomonas	UA|UAn
Bacteria; Cyanobacteria; Unclassified Cyanobacteria; Nostocales; Nostocaceae; Nostoc	UA|UAn
Bacteria; Proteobacteria; Gammaproteobacteria; Chromatiales; Thioprofundaceae; Thioprofundum	UA|UAn
Bacteria; Proteobacteria; Alphaproteobacteria; Rhodospirillales; Acetobacteraceae; Acidisoma	UA|UAn
Bacteria; Proteobacteria; Gammaproteobacteria; Legionellales; Legionellaceae; Legionella	UA|UAn
Bacteria; Verrucomicrobia; Verrucomicrobiae; Verrucomicrobiales; Verrucomicrobiaceae; Luteolibacter	UA|UAn
Bacteria; Proteobacteria; Betaproteobacteria; Nitrosomonadales; Nitrosomonadaceae; Nitrosospira	UA|UAn
Bacteria; Proteobacteria; Betaproteobacteria; Rhodocyclales; Rhodocyclaceae; Propionivibrio	UA|UAn
Bacteria; Actinobacteria; Actinomycetia; Streptosporangiales; Thermomonosporaceae; Actinocorallia	UA|UAn
Bacteria; Proteobacteria; Oligoflexia; Bdellovibrionales; Bdellovibrionaceae; Bdellovibrio	UA|UAn
Bacteria; Proteobacteria; Betaproteobacteria; Burkholderiales; Unclassified Burkholderiales; Piscinibacterium	UA|UAn
Bacteria; Proteobacteria; Deltaproteobacteria; Myxococcales; Myxococcaceae; Myxococcus	UA|UAn
Bacteria; Bacteroidetes; Cytophagia; Cytophagales; Fulvivirgaceae; Ohtaekwangia	UA|UAn
Bacteria; Proteobacteria; Gammaproteobacteria; Enterobacterales; Enterobacteriaceae; Kluyvera	UA|UAn
Bacteria; Chloroflexi; Thermomicrobia; Thermomicrobiales; Thermomicrobiaceae; Thermomicrobium	UA|UAn
Bacteria; Proteobacteria; Deltaproteobacteria; Myxococcales; Kofleriaceae; Kofleria	UA|UAn
Bacteria; Proteobacteria; Betaproteobacteria; Burkholderiales; Oxalobacteraceae; Massilia	UA|UAn
Bacteria; Actinobacteria; Actinomycetia; Streptosporangiales; Streptosporangiaceae; Sphaerisporangium	UA|UAn
Bacteria; Proteobacteria; Deltaproteobacteria; Desulfuromonadales; Syntrophotaleaceae; Syntrophotalea	UA|UAn
Bacteria; Proteobacteria; Alphaproteobacteria; Rhodospirillales; Acetobacteraceae; Rhodopila	UA|UAn
Bacteria; Actinobacteria; Actinomycetia; Micromonosporales; Micromonosporaceae; Dactylosporangium	UA|UAn
Bacteria; Proteobacteria; Alphaproteobacteria; Micropepsales; Micropepsaceae; Micropepsis	UA|UAn
Bacteria; Actinobacteria; Acidimicrobiia; Acidimicrobiales; Acidimicrobiaceae; Acidiferrimicrobium	UA|UAn
Bacteria; Bacteroidetes; Chitinophagia; Chitinophagales; Chitinophagaceae; Flavitalea	UA|UAn
Bacteria; Planctomycetes; Planctomycetia; Gemmatales; Gemmataceae; Gemmata	UA|UAn
Bacteria; Proteobacteria; Betaproteobacteria; Nitrosomonadales; Sterolibacteriaceae; Sulfurisoma	UA|UAn
Bacteria; Proteobacteria; Alphaproteobacteria; Hyphomicrobiales; Brucellaceae; Pseudochrobactrum	UA|UAn
Bacteria; Bacteroidetes; Sphingobacteriia; Sphingobacteriales; Sphingobacteriaceae; Pararcticibacter	UA|UAn
Bacteria; Proteobacteria; Betaproteobacteria; Burkholderiales; Alcaligenaceae; Azohydromonas	UA|UAn
Bacteria; Proteobacteria; Alphaproteobacteria; Hyphomicrobiales; Rhodobiaceae; Rhodobium	UA|UAn
Bacteria; Proteobacteria; Betaproteobacteria; Burkholderiales; Unclassified Burkholderiales; Thiobacter	UA|UAn
Bacteria; Acidobacteria; Blastocatellia; Blastocatellales; Blastocatellaceae; Tellurimicrobium	UA|UAn
Bacteria; Bacteroidetes; Chitinophagia; Chitinophagales; Chitinophagaceae; Ferruginibacter	UA|UAn
Bacteria; Firmicutes; Bacilli; Bacillales; Bacillaceae; Psychrobacillus	UA|UAn
Bacteria; Deinococcus-Thermus; Deinococci; Trueperales; Trueperaceae; Truepera	UA|UAn
Bacteria; Proteobacteria; Alphaproteobacteria; Hyphomicrobiales; Methylobacteriaceae; Methylobacterium	UA|UAn
Bacteria; Proteobacteria; Betaproteobacteria; Burkholderiales; Burkholderiaceae; Robbsia	UA|UAn
Bacteria; Chloroflexi; Ardenticatenia; Ardenticatenales; Ardenticatenaceae; Ardenticatena	UA|UAn
Bacteria; Planctomycetes; Planctomycetia; Isosphaerales; Isosphaeraceae; Aquisphaera	UA|UAn
Bacteria; Bacteroidetes; Chitinophagia; Chitinophagales; Chitinophagaceae; Segetibacter	UA|UAn
Bacteria; Actinobacteria; Actinomycetia; Pseudonocardiales; Pseudonocardiaceae; Salinifilum	UA|UAn
Bacteria; Firmicutes; Bacilli; Bacillales; Bacillaceae; Litchfieldia	UA|UAn
Bacteria; Actinobacteria; Actinomycetia; Micrococcales; Promicromonosporaceae; Cellulosimicrobium	UA|UAn
Bacteria; Proteobacteria; Gammaproteobacteria; Pseudomonadales; Moraxellaceae; Psychrobacter	UA|UAn
Bacteria; Gemmatimonadetes; Gemmatimonadetes; Gemmatimonadales; Gemmatimonadaceae; Gemmatimonas	UA|UAn
Bacteria; Actinobacteria; Acidimicrobiia; Acidimicrobiales; Iamiaceae; Iamia	UA|UAn
Bacteria; Actinobacteria; Actinomycetia; Propionibacteriales; Nocardioidaceae; Marmoricola	UA|UAn
Bacteria; Proteobacteria; Alphaproteobacteria; Rhodospirillales; Azospirillaceae; Niveispirillum	UA|UAn
Bacteria; Acidobacteria; Acidobacteriia; Acidobacteriales; Acidobacteriaceae; Telmatobacter	UA|UAn
Bacteria; Firmicutes; Bacilli; Bacillales; Bacillaceae; Quasibacillus	UA|UAn
Bacteria; Chlamydiae; Chlamydiia; Parachlamydiales; Parachlamydiaceae; Parachlamydia	UA|UAn
Bacteria; Actinobacteria; Actinomycetia; Micrococcales; Microbacteriaceae; Homoserinibacter	UA|UAn
Bacteria; Gemmatimonadetes; Longimicrobia; Longimicrobiales; Longimicrobiaceae; Longimicrobium	UA|UAn
Bacteria; Proteobacteria; Betaproteobacteria; Nitrosomonadales; Sterolibacteriaceae; Denitratisoma	UA|UAn
Bacteria; Planctomycetes; Planctomycetia; Pirellulales; Thermoguttaceae; Thermostilla	UA|UAn
Bacteria; Proteobacteria; Deltaproteobacteria; Myxococcales; Archangiaceae; Cystobacter	UA|UAn
Bacteria; Proteobacteria; Gammaproteobacteria; Legionellales; Coxiellaceae; Coxiella	UA|UAn
Bacteria; Planctomycetes; Planctomycetia; Planctomycetales; Planctomycetaceae; Planctopirus	UA|UAn
Bacteria; Actinobacteria; Actinomycetia; Propionibacteriales; Kribbellaceae; Kribbella	UA|UAn

Bacteria; Bacteroidetes; Flavobacteriia; Flavobacteriales; Weeksellaceae; Chryseobacterium	UA|UAn
