# Supplementary material for: Composition and functional diversity of bacterial communities during swine carcass decomposition
Source: Anim Biosci. 2023 Jun 26;36(9):1453–64. doi: 10.5713/ab.23.0140 (PMC10472150; doi:10.5713/ab.23.0140)
Supplement: Supplementary file 4 [file ab-23-0140-Supplementary-Table-3.pdf]

Table S3. Taxonomic classification of shared bacterial genera present in UA, SA, UAn, and SAn samples

<table>
<tr><th>Taxonomy</th><th>Burial set-up</th></tr>
<tr><td>Bacteria; Firmicutes; Erysipelotrichia; Erysipelotrichales; Erysipelotrichaceae; Solobacterium</td><td>UA|SA|UAn|SAn</td></tr>
<tr><td>Bacteria; Firmicutes; Bacilli; Bacillales; Bacillaceae; Paucisalibacillus</td><td>UA|SA|UAn|SAn</td></tr>
<tr><td>Bacteria; Firmicutes; Tissierellia; Tissierellales; Tissierellaceae; Tissierella</td><td>UA|SA|UAn|SAn</td></tr>
<tr><td>Bacteria; Actinobacteria; Coriobacteriia; Coriobacteriales; Coriobacteriaceae; Senegalimassilia</td><td>UA|SA|UAn|SAn</td></tr>
<tr><td>Bacteria; Firmicutes; Bacilli; Bacillales; Paenibacillaceae; Marinicrinis</td><td>UA|SA|UAn|SAn</td></tr>
<tr><td>Bacteria; Firmicutes; Clostridia; Eubacteriales; Oscillospiraceae; Anaerotruncus</td><td>UA|SA|UAn|SAn</td></tr>
<tr><td>Bacteria; Firmicutes; Bacilli; Bacillales; Alicyclobacillaceae; Tumebacillus</td><td>UA|SA|UAn|SAn</td></tr>
<tr><td>Bacteria; Firmicutes; Bacilli; Bacillales; Thermoactinomycetaceae; Thermoactinomyces</td><td>UA|SA|UAn|SAn</td></tr>
<tr><td>Bacteria; Proteobacteria; Gammaproteobacteria; Enterobacterales; Enterobacteriaceae; Pseudescherichia</td><td>UA|SA|UAn|SAn</td></tr>
<tr><td>Bacteria; Firmicutes; Bacilli; Lactobacillales; Lactobacillaceae; Levilactobacillus</td><td>UA|SA|UAn|SAn</td></tr>
<tr><td>Bacteria; Firmicutes; Clostridia; Eubacteriales; Clostridiales Family XIII. Incertae Sedis; Mogibacterium</td><td>UA|SA|UAn|SAn</td></tr>
<tr><td>Bacteria; Firmicutes; Bacilli; Lactobacillales; Lactobacillaceae; Companilactobacillus</td><td>UA|SA|UAn|SAn</td></tr>
<tr><td>Bacteria; Firmicutes; Bacilli; Bacillales; Staphylococcaceae; Staphylococcus</td><td>UA|SA|UAn|SAn</td></tr>
<tr><td>Bacteria; Firmicutes; Clostridia; Eubacteriales; Clostridiaceae; Geosporobacter</td><td>UA|SA|UAn|SAn</td></tr>
<tr><td>Bacteria; Proteobacteria; Gammaproteobacteria; Pasteurellales; Pasteurellaceae; Actinobacillus</td><td>UA|SA|UAn|SAn</td></tr>
<tr><td>Bacteria; Firmicutes; Clostridia; Eubacteriales; Oscillospiraceae; Ruminiclostridium</td><td>UA|SA|UAn|SAn</td></tr>
<tr><td>Bacteria; Bacteroidetes; Bacteroidia; Bacteroidales; Muribaculaceae; Muribaculum</td><td>UA|SA|UAn|SAn</td></tr>
<tr><td>Bacteria; Firmicutes; Bacilli; Bacillales; Planococcaceae; Kurthia</td><td>UA|SA|UAn|SAn</td></tr>
<tr><td>Bacteria; Firmicutes; Clostridia; Eubacteriales; Oscillospiraceae; Ruminococcus</td><td>UA|SA|UAn|SAn</td></tr>
<tr><td>Bacteria; Firmicutes; Clostridia; Eubacteriales; Proteinivoraceae; Anaerobranca</td><td>UA|SA|UAn|SAn</td></tr>
<tr><td>Bacteria; Actinobacteria; Coriobacteriia; Coriobacteriales; Atopobiaceae; Olsenella</td><td>UA|SA|UAn|SAn</td></tr>
<tr><td>Bacteria; Firmicutes; Bacilli; Lactobacillales; Streptococcaceae; Lactococcus</td><td>UA|SA|UAn|SAn</td></tr>
<tr><td>Bacteria; Firmicutes; Clostridia; Eubacteriales; Vallitaleaceae; Vallitalea</td><td>UA|SA|UAn|SAn</td></tr>
<tr><td>Bacteria; Firmicutes; Bacilli; Bacillales; Bacillaceae; Gracilibacillus</td><td>UA|SA|UAn|SAn</td></tr>
<tr><td>Bacteria; Firmicutes; Bacilli; Bacillales; Bacillaceae; Ornithinibacillus</td><td>UA|SA|UAn|SAn</td></tr>
<tr><td>Bacteria; Firmicutes; Clostridia; Eubacteriales; Peptostreptococcaceae; Paraclostridium</td><td>UA|SA|UAn|SAn</td></tr>
<tr><td>Bacteria; Firmicutes; Erysipelotrichia; Erysipelotrichales; Coprobacillaceae; Sharpea</td><td>UA|SA|UAn|SAn</td></tr>
<tr><td>Bacteria; Firmicutes; Unclassified Firmicutes; Unclassified Firmicutes; Unclassified Firmicutes; Hydrogenispora</td><td>UA|SA|UAn|SAn</td></tr>
<tr><td>Bacteria; Firmicutes; Clostridia; Eubacteriales; Peptococcaceae; Desulfallas</td><td>UA|SA|UAn|SAn</td></tr>
<tr><td>Bacteria; Firmicutes; Negativicutes; Acidaminococcales; Acidaminococcaceae; Phascolarctobacterium</td><td>UA|SA|UAn|SAn</td></tr>
<tr><td>Bacteria; Firmicutes; Clostridia; Eubacteriales; Oscillospiraceae; Faecalibacterium</td><td>UA|SA|UAn|SAn</td></tr>
<tr><td>Bacteria; Actinobacteria; Coriobacteriia; Coriobacteriales; Coriobacteriaceae; Parvibacter</td><td>UA|SA|UAn|SAn</td></tr>
<tr><td>Bacteria; Acidobacteria; Acidobacteriia; Acidobacteriales; Acidobacteriaceae; Acidobacterium</td><td>UA|SA|UAn|SAn</td></tr>
<tr><td>Bacteria; Firmicutes; Bacilli; Bacillales; Bacillaceae; Lederbergia</td><td>UA|SA|UAn|SAn</td></tr>
<tr><td>Bacteria; Actinobacteria; Actinomycetia; Streptosporangiales; Streptosporangiaceae; Nonomuraea</td><td>UA|SA|UAn|SAn</td></tr>
<tr><td>Bacteria; Firmicutes; Bacilli; Bacillales; Bacillaceae; Halobacillus</td><td>UA|SA|UAn|SAn</td></tr>
<tr><td>Bacteria; Firmicutes; Bacilli; Lactobacillales; Lactobacillaceae; Pediococcus</td><td>UA|SA|UAn|SAn</td></tr>
<tr><td>Bacteria; Firmicutes; Clostridia; Eubacteriales; Clostridiales Family XIII. Incertae Sedis; Ihubacter</td><td>UA|SA|UAn|SAn</td></tr>
<tr><td>Bacteria; Firmicutes; Tissierellia; Tissierellales; Tissierellaceae; Anaerosalibacter</td><td>UA|SA|UAn|SAn</td></tr>
<tr><td>Bacteria; Firmicutes; Erysipelotrichia; Erysipelotrichales; Coprobacillaceae; Catenibacterium</td><td>UA|SA|UAn|SAn</td></tr>
<tr><td>Bacteria; Firmicutes; Bacilli; Lactobacillales; Enterococcaceae; Enterococcus</td><td>UA|SA|UAn|SAn</td></tr>
<tr><td>Bacteria; Firmicutes; Clostridia; Eubacteriales; Oscillospiraceae; Unclassified Oscillospiraceae</td><td>UA|SA|UAn|SAn</td></tr>
<tr><td>Bacteria; Firmicutes; Erysipelotrichia; Erysipelotrichales; Erysipelotrichaceae; Holdemania</td><td>UA|SA|UAn|SAn</td></tr>
<tr><td>Bacteria; Firmicutes; Bacilli; Bacillales; Unclassified Bacillales; Unclassified Bacillales</td><td>UA|SA|UAn|SAn</td></tr>
<tr><td>Bacteria; Firmicutes; Bacilli; Bacillales; Bacillaceae; Heyndrickxia</td><td>UA|SA|UAn|SAn</td></tr>
<tr><td>Bacteria; Bacteroidetes; Bacteroidia; Bacteroidales; Prevotellaceae; Alloprevotella</td><td>UA|SA|UAn|SAn</td></tr>
<tr><td>Bacteria; Firmicutes; Negativicutes; Veillonellales; Veillonellaceae; Veillonella</td><td>UA|SA|UAn|SAn</td></tr>
<tr><td>Bacteria; Firmicutes; Erysipelotrichia; Erysipelotrichales; Turicibacteraceae; Turicibacter</td><td>UA|SA|UAn|SAn</td></tr>
<tr><td>Bacteria; Firmicutes; Clostridia; Eubacteriales; Lachnospiraceae; Fusicatenibacter</td><td>UA|SA|UAn|SAn</td></tr>
<tr><td>Bacteria; Actinobacteria; Actinomycetia; Geodermatophilales; Geodermatophilaceae; Blastococcus</td><td>UA|SA|UAn|SAn</td></tr>
<tr><td>Bacteria; Firmicutes; Clostridia; Eubacteriales; Clostridiaceae; Clostridium</td><td>UA|SA|UAn|SAn</td></tr>
<tr><td>Bacteria; Firmicutes; Negativicutes; Acidaminococcales; Acidaminococcaceae; Acidaminococcus</td><td>UA|SA|UAn|SAn</td></tr>
<tr><td>Bacteria; Firmicutes; Bacilli; Bacillales; Planococcaceae; Ureibacillus</td><td>UA|SA|UAn|SAn</td></tr>
<tr><td>Bacteria; Firmicutes; Clostridia; Eubacteriales; Oscillospiraceae; Fournierella</td><td>UA|SA|UAn|SAn</td></tr>
<tr><td>Bacteria; Proteobacteria; Gammaproteobacteria; Pseudomonadales; Pseudomonadaceae; Pseudomonas</td><td>UA|SA|UAn|SAn</td></tr>
<tr><td>Bacteria; Firmicutes; Clostridia; Eubacteriales; Lachnospiraceae; Blautia</td><td>UA|SA|UAn|SAn</td></tr>
<tr><td>Bacteria; Firmicutes; Clostridia; Thermoanaerobacterales; Thermoanaerobacterales Family III. Incertae Sedis; Syntrophaceticus</td><td>UA|SA|UAn|SAn</td></tr>
<tr><td>Bacteria; Firmicutes; Clostridia; Eubacteriales; Lachnospiraceae; Coprococcus</td><td>UA|SA|UAn|SAn</td></tr>
<tr><td>Bacteria; Firmicutes; Clostridia; Eubacteriales; Oscillospiraceae; Caproiciproducens</td><td>UA|SA|UAn|SAn</td></tr>
<tr><td>Bacteria; Firmicutes; Clostridia; Eubacteriales; Clostridiaceae; Sporosalibacterium</td><td>UA|SA|UAn|SAn</td></tr>
<tr><td>Bacteria; Firmicutes; Bacilli; Lactobacillales; Lactobacillaceae; Weissella</td><td>UA|SA|UAn|SAn</td></tr>
<tr><td>Bacteria; Firmicutes; Clostridia; Eubacteriales; Unclassified Eubacteriales; Colidextribacter</td><td>UA|SA|UAn|SAn</td></tr>
<tr><td>Bacteria; Actinobacteria; Coriobacteriia; Eggerthellales; Eggerthellaceae; Adlercreutzia</td><td>UA|SA|UAn|SAn</td></tr>
<tr><td>Bacteria; Firmicutes; Clostridia; Eubacteriales; Lachnospiraceae; Oribacterium</td><td>UA|SA|UAn|SAn</td></tr>
<tr><td>Bacteria; Actinobacteria; Coriobacteriia; Coriobacteriales; Coriobacteriaceae; Collinsella</td><td>UA|SA|UAn|SAn</td></tr>
<tr><td>Bacteria; Firmicutes; Bacilli; Lactobacillales; Lactobacillaceae; Lentilactobacillus</td><td>UA|SA|UAn|SAn</td></tr>
<tr><td>Bacteria; Firmicutes; Clostridia; Thermosediminibacterales; Tepidanaerobacteraceae; Tepidanaerobacter</td><td>UA|SA|UAn|SAn</td></tr>
<tr><td>Bacteria; Firmicutes; Bacilli; Lactobacillales; Lactobacillaceae; Agrilactobacillus</td><td>UA|SA|UAn|SAn</td></tr>
<tr><td>Bacteria; Firmicutes; Erysipelotrichia; Erysipelotrichales; Erysipelotrichaceae; Erysipelothrix</td><td>UA|SA|UAn|SAn</td></tr>
<tr><td>Bacteria; Firmicutes; Erysipelotrichia; Erysipelotrichales; Erysipelotrichaceae; Holdemanella</td><td>UA|SA|UAn|SAn</td></tr>
<tr><td>Bacteria; Firmicutes; Bacilli; Bacillales; Bacillaceae; Niallia</td><td>UA|SA|UAn|SAn</td></tr>
<tr><td>Bacteria; Actinobacteria; Coriobacteriia; Eggerthellales; Eggerthellaceae; Eggerthella</td><td>UA|SA|UAn|SAn</td></tr>
<tr><td>Bacteria; Actinobacteria; Actinomycetia; Corynebacteriales; Corynebacteriaceae; Corynebacterium</td><td>UA|SA|UAn|SAn</td></tr>
<tr><td>Bacteria; Proteobacteria; Deltaproteobacteria; Myxococcales; Labilitrichaceae; Labilithrix</td><td>UA|SA|UAn|SAn</td></tr>
<tr><td>Bacteria; Actinobacteria; Actinomycetia; Streptosporangiales; Streptosporangiaceae; Microbispora</td><td>UA|SA|UAn|SAn</td></tr>
<tr><td>Bacteria; Firmicutes; Clostridia; Eubacteriales; Oscillospiraceae; Oscillibacter</td><td>UA|SA|UAn|SAn</td></tr>
<tr><td>Bacteria; Actinobacteria; Coriobacteriia; Coriobacteriales; Coriobacteriaceae; Enorma</td><td>UA|SA|UAn|SAn</td></tr>
<tr><td>Bacteria; Firmicutes; Clostridia; Eubacteriales; Lachnospiraceae; Anaerotaenia</td><td>UA|SA|UAn|SAn</td></tr>
<tr><td>Bacteria; Actinobacteria; Actinomycetia; Propionibacteriales; Nocardioidaceae; Nocardioides</td><td>UA|SA|UAn|SAn</td></tr>
<tr><td>Bacteria; Firmicutes; Negativicutes; Selenomonadales; Selenomonadaceae; Mitsuokella</td><td>UA|SA|UAn|SAn</td></tr>
<tr><td>Bacteria; Firmicutes; Clostridia; Eubacteriales; Unclassified Eubacteriales; Proteiniborus</td><td>UA|SA|UAn|SAn</td></tr>
<tr><td>Bacteria; Bacteroidetes; Bacteroidia; Bacteroidales; Unclassified Bacteroidales; Phocaeicola</td><td>UA|SA|UAn|SAn</td></tr>
<tr><td>Bacteria; Firmicutes; Clostridia; Eubacteriales; Lachnospiraceae; Cellulosilyticum</td><td>UA|SA|UAn|SAn</td></tr>
<tr><td>Bacteria; Firmicutes; Tissierellia; Tissierellales; Tissierellaceae; Urmitella</td><td>UA|SA|UAn|SAn</td></tr>
<tr><td>Bacteria; Proteobacteria; Alphaproteobacteria; Hyphomicrobiales; Phyllobacteriaceae; Mesorhizobium</td><td>UA|SA|UAn|SAn</td></tr>
<tr><td>Bacteria; Firmicutes; Bacilli; Bacillales; Bacillaceae; Caldibacillus</td><td>UA|SA|UAn|SAn</td></tr>
<tr><td>Bacteria; Firmicutes; Clostridia; Eubacteriales; Peptococcaceae; Desulfitibacter</td><td>UA|SA|UAn|SAn</td></tr>
<tr><td>Bacteria; Firmicutes; Clostridia; Eubacteriales; Christensenellaceae; Christensenella</td><td>UA|SA|UAn|SAn</td></tr>
<tr><td>Bacteria; Firmicutes; Clostridia; Eubacteriales; Peptostreptococcaceae; Romboutsia</td><td>UA|SA|UAn|SAn</td></tr>
<tr><td>Bacteria; Proteobacteria; Deltaproteobacteria; Desulfovibrionales; Desulfovibrionaceae; Desulfovibrio</td><td>UA|SA|UAn|SAn</td></tr>
<tr><td>Bacteria; Firmicutes; Bacilli; Bacillales; Bacillaceae; Oceanobacillus</td><td>UA|SA|UAn|SAn</td></tr>
<tr><td>Bacteria; Firmicutes; Bacilli; Bacillales; Bacillaceae; Sinibacillus</td><td>UA|SA|UAn|SAn</td></tr>
<tr><td>Bacteria; Firmicutes; Clostridia; Eubacteriales; Oscillospiraceae; Ruthenibacterium</td><td>UA|SA|UAn|SAn</td></tr>
<tr><td>Bacteria; Firmicutes; Clostridia; Eubacteriales; Oscillospiraceae; Anaerobacterium</td><td>UA|SA|UAn|SAn</td></tr>
<tr><td>Bacteria; Firmicutes; Clostridia; Eubacteriales; Lachnospiraceae; Anaerobutyricum</td><td>UA|SA|UAn|SAn</td></tr>
<tr><td>Bacteria; Firmicutes; Clostridia; Eubacteriales; Clostridiaceae; Maledivibacter</td><td>UA|SA|UAn|SAn</td></tr>
<tr><td>Bacteria; Firmicutes; Bacilli; Bacillales; Planococcaceae; Bhargavaea</td><td>UA|SA|UAn|SAn</td></tr>
<tr><td>Bacteria; Firmicutes; Bacilli; Bacillales; Bacillaceae; Rossellomorea</td><td>UA|SA|UAn|SAn</td></tr>
<tr><td>Bacteria; Firmicutes; Clostridia; Eubacteriales; Lachnospiraceae; Lachnoclostridium</td><td>UA|SA|UAn|SAn</td></tr>
<tr><td>Bacteria; Firmicutes; Clostridia; Eubacteriales; Clostridiales Family XIII. Incertae Sedis; Anaerovorax</td><td>UA|SA|UAn|SAn</td></tr>
<tr><td>Bacteria; Actinobacteria; Actinomycetia; Corynebacteriales; Mycobacteriaceae; Mycolicibacterium</td><td>UA|SA|UAn|SAn</td></tr>
<tr><td>Bacteria; Firmicutes; Bacilli; Lactobacillales; Enterococcaceae; Vagococcus</td><td>UA|SA|UAn|SAn</td></tr>
<tr><td>Bacteria; Actinobacteria; Actinomycetia; Micromonosporales; Micromonosporaceae; Micromonospora</td><td>UA|SA|UAn|SAn</td></tr>
<tr><td>Bacteria; Firmicutes; Bacilli; Bacillales; Thermoactinomycetaceae; Novibacillus</td><td>UA|SA|UAn|SAn</td></tr>
<tr><td>Bacteria; Firmicutes; Clostridia; Eubacteriales; Clostridiales Family XIII. Incertae Sedis; Emergencia</td><td>UA|SA|UAn|SAn</td></tr>
<tr><td>Bacteria; Actinobacteria; Actinomycetia; Streptosporangiales; Thermomonosporaceae; Actinomadura</td><td>UA|SA|UAn|SAn</td></tr>
<tr><td>Bacteria; Firmicutes; Clostridia; Eubacteriales; Eubacteriaceae; Eubacterium</td><td>UA|SA|UAn|SAn</td></tr>
<tr><td>Bacteria; Firmicutes; Clostridia; Eubacteriales; Lachnospiraceae; Unclassified Lachnospiraceae</td><td>UA|SA|UAn|SAn</td></tr>
<tr><td>Bacteria; Firmicutes; Clostridia; Eubacteriales; Eubacteriaceae; Garciella</td><td>UA|SA|UAn|SAn</td></tr>
<tr><td>Bacteria; Planctomycetes; Planctomycetia; Pirellulales; Lacipirellulaceae; Bythopirellula</td><td>UA|SA|UAn|SAn</td></tr>
<tr><td>Bacteria; Firmicutes; Clostridia; Eubacteriales; Peptostreptococcaceae; Peptostreptococcus</td><td>UA|SA|UAn|SAn</td></tr>
<tr><td>Bacteria; Firmicutes; Bacilli; Bacillales; Bacillaceae; Gottfriedia</td><td>UA|SA|UAn|SAn</td></tr>
<tr><td>Bacteria; Firmicutes; Limnochordia; Limnochordales; Limnochordaceae; Limnochorda</td><td>UA|SA|UAn|SAn</td></tr>
<tr><td>Bacteria; Firmicutes; Bacilli; Bacillales; Bacillaceae; Sutcliffiella</td><td>UA|SA|UAn|SAn</td></tr>
</table>

<table>
<tr><td>Bacteria; Firmicutes; Erysipelotrichia; Erysipelotrichales; Erysipelotrichaceae; Faecalitalea</td><td>UA|SA|UAn|SAn</td></tr>
<tr><td>Bacteria; Proteobacteria; Alphaproteobacteria; Hyphomicrobiales; Bradyrhizobiaceae; Bradyrhizobium</td><td>UA|SA|UAn|SAn</td></tr>
<tr><td>Bacteria; Firmicutes; Bacilli; Bacillales; Bacillaceae; Cerasibacillus</td><td>UA|SA|UAn|SAn</td></tr>
<tr><td>Bacteria; Proteobacteria; Alphaproteobacteria; Hyphomicrobiales; Xanthobacteraceae; Pseudolabrys</td><td>UA|SA|UAn|SAn</td></tr>
<tr><td>Bacteria; Firmicutes; Bacilli; Bacillales; Bacillaceae; Neobacillus</td><td>UA|SA|UAn|SAn</td></tr>
<tr><td>Bacteria; Firmicutes; Clostridia; Eubacteriales; Peptostreptococcaceae; Terrisporobacter</td><td>UA|SA|UAn|SAn</td></tr>
<tr><td>Bacteria; Firmicutes; Tissierellia; Tissierellales; Tissierellaceae; Sporanaerobacter</td><td>UA|SA|UAn|SAn</td></tr>
<tr><td>Bacteria; Firmicutes; Bacilli; Bacillales; Thermoactinomycetaceae; Planifilum</td><td>UA|SA|UAn|SAn</td></tr>
<tr><td>Bacteria; Firmicutes; Tissierellia; Tissierellales; Peptoniphilaceae; Peptoniphilus</td><td>UA|SA|UAn|SAn</td></tr>
<tr><td>Bacteria; Actinobacteria; Actinomycetia; Micromonosporales; Micromonosporaceae; Rugosimonospora</td><td>UA|SA|UAn|SAn</td></tr>
<tr><td>Bacteria; Firmicutes; Tissierellia; Unclassified Tissierellia; Unclassified Tissierellia; Sedimentibacter</td><td>UA|SA|UAn|SAn</td></tr>
<tr><td>Bacteria; Bacteroidetes; Bacteroidia; Bacteroidales; Prevotellaceae; Prevotella</td><td>UA|SA|UAn|SAn</td></tr>
<tr><td>Bacteria; Firmicutes; Bacilli; Bacillales; Bacillaceae; Caldalkalibacillus</td><td>UA|SA|UAn|SAn</td></tr>
<tr><td>Bacteria; Firmicutes; Clostridia; Eubacteriales; Lachnospiraceae; Lacrimispora</td><td>UA|SA|UAn|SAn</td></tr>
<tr><td>Bacteria; Firmicutes; Bacilli; Bacillales; Bacillaceae; Alkalihalobacillus</td><td>UA|SA|UAn|SAn</td></tr>
<tr><td>Bacteria; Acidobacteria; Acidobacteriia; Acidobacteriales; Acidobacteriaceae; Occallatibacter</td><td>UA|SA|UAn|SAn</td></tr>
<tr><td>Bacteria; Firmicutes; Bacilli; Bacillales; Bacillaceae; Virgibacillus</td><td>UA|SA|UAn|SAn</td></tr>
<tr><td>Bacteria; Firmicutes; Clostridia; Eubacteriales; Oscillospiraceae; Acetanaerobacterium</td><td>UA|SA|UAn|SAn</td></tr>
<tr><td>Bacteria; Firmicutes; Clostridia; Eubacteriales; Caldicoprobacteraceae; Caldicoprobacter</td><td>UA|SA|UAn|SAn</td></tr>
<tr><td>Bacteria; Firmicutes; Bacilli; Bacillales; Bacillaceae; Bacillus</td><td>UA|SA|UAn|SAn</td></tr>
<tr><td>Bacteria; Firmicutes; Clostridia; Eubacteriales; Symbiobacteriaceae; Symbiobacterium</td><td>UA|SA|UAn|SAn</td></tr>
<tr><td>Bacteria; Firmicutes; Bacilli; Bacillales; Thermoactinomycetaceae; Shimazuella</td><td>UA|SA|UAn|SAn</td></tr>
<tr><td>Bacteria; Firmicutes; Clostridia; Eubacteriales; Unclassified Eubacteriales; Gemmiger</td><td>UA|SA|UAn|SAn</td></tr>
<tr><td>Bacteria; Firmicutes; Clostridia; Natranaerobiales; Natranaerobiaceae; Natranaerobaculum</td><td>UA|SA|UAn|SAn</td></tr>
<tr><td>Bacteria; Proteobacteria; Alphaproteobacteria; Hyphomicrobiales; Devosiaceae; Devosia</td><td>UA|SA|UAn|SAn</td></tr>
<tr><td>Bacteria; Firmicutes; Clostridia; Eubacteriales; Clostridiaceae; Haloimpatiens</td><td>UA|SA|UAn|SAn</td></tr>
<tr><td>Bacteria; Firmicutes; Tissierellia; Tissierellales; Tissierellaceae; Tepidimicrobium</td><td>UA|SA|UAn|SAn</td></tr>
<tr><td>Bacteria; Bacteroidetes; Bacteroidia; Bacteroidales; Barnesiellaceae; Barnesiella</td><td>UA|SA|UAn|SAn</td></tr>
<tr><td>Bacteria; Firmicutes; Clostridia; Eubacteriales; Lachnospiraceae; Lachnospira</td><td>UA|SA|UAn|SAn</td></tr>
<tr><td>Bacteria; Firmicutes; Clostridia; Eubacteriales; Lachnospiraceae; Enterocloster</td><td>UA|SA|UAn|SAn</td></tr>
<tr><td>Bacteria; Bacteroidetes; Bacteroidia; Bacteroidales; Muribaculaceae; Duncaniella</td><td>UA|SA|UAn|SAn</td></tr>
<tr><td>Bacteria; Proteobacteria; Gammaproteobacteria; Pasteurellales; Pasteurellaceae; UnclassifiedPasteurellaceae</td><td>UA|SA|UAn|SAn</td></tr>
<tr><td>Bacteria; Firmicutes; Tissierellia; Tissierellales; Tissierellaceae; Schnuerera</td><td>UA|SA|UAn|SAn</td></tr>
<tr><td>Bacteria; Firmicutes; Clostridia; Eubacteriales; Clostridiaceae; Lutispora</td><td>UA|SA|UAn|SAn</td></tr>
<tr><td>Bacteria; Firmicutes; Clostridia; Eubacteriales; Lachnospiraceae; Murimonas</td><td>UA|SA|UAn|SAn</td></tr>
<tr><td>Bacteria; Cyanobacteria; Unclassified Cyanobacteria; Oscillatoriales; Oscillatoriaceae; Aerosakkonema</td><td>UA|SA|UAn|SAn</td></tr>
<tr><td>Bacteria; Firmicutes; Bacilli; Bacillales; Paenibacillaceae; Ammoniphilus</td><td>UA|SA|UAn|SAn</td></tr>
<tr><td>Bacteria; Actinobacteria; Actinomycetia; Micromonosporales; Micromonosporaceae; Actinoplanes</td><td>UA|SA|UAn|SAn</td></tr>
<tr><td>Bacteria; Firmicutes; Clostridia; Eubacteriales; Lachnospiraceae; Butyrivibrio</td><td>UA|SA|UAn|SAn</td></tr>
<tr><td>Bacteria; Proteobacteria; Gammaproteobacteria; Enterobacterales; Morganellaceae; Proteus</td><td>UA|SA|UAn|SAn</td></tr>
<tr><td>Bacteria; Firmicutes; Bacilli; Bacillales; Paenibacillaceae; Paenibacillus</td><td>UA|SA|UAn|SAn</td></tr>
<tr><td>Bacteria; Firmicutes; Clostridia; Eubacteriales; Lachnospiraceae; Dorea</td><td>UA|SA|UAn|SAn</td></tr>
<tr><td>Bacteria; Actinobacteria; Actinomycetia; Streptomycetales; Streptomycetaceae; Kitasatospora</td><td>UA|SA|UAn|SAn</td></tr>
<tr><td>Bacteria; Firmicutes; Clostridia; Eubacteriales; Peptostreptococcaceae; Paeniclostridium</td><td>UA|SA|UAn|SAn</td></tr>
<tr><td>Bacteria; Firmicutes; Clostridia; Eubacteriales; Peptococcaceae; Peptococcus</td><td>UA|SA|UAn|SAn</td></tr>
<tr><td>Bacteria; Firmicutes; Bacilli; Bacillales; Bacillaceae; Pseudogracilibacillus</td><td>UA|SA|UAn|SAn</td></tr>
<tr><td>Bacteria; Firmicutes; Bacilli; Bacillales; Bacillaceae; Fictibacillus</td><td>UA|SA|UAn|SAn</td></tr>
<tr><td>Bacteria; Firmicutes; Clostridia; Eubacteriales; Unclassified Eubacteriales; Intestinimonas</td><td>UA|SA|UAn|SAn</td></tr>
<tr><td>Bacteria; Firmicutes; Bacilli; Bacillales; Bacillaceae; Alkalicoccus</td><td>UA|SA|UAn|SAn</td></tr>
<tr><td>Bacteria; Firmicutes; Bacilli; Lactobacillales; Lactobacillaceae; Ligilactobacillus</td><td>UA|SA|UAn|SAn</td></tr>
<tr><td>Bacteria; Firmicutes; Clostridia; Eubacteriales; Lachnospiraceae; Mediterraneibacter</td><td>UA|SA|UAn|SAn</td></tr>
<tr><td>Bacteria; Firmicutes; Bacilli; Bacillales; Bacillaceae; Peribacillus</td><td>UA|SA|UAn|SAn</td></tr>
<tr><td>Bacteria; Firmicutes; Clostridia; Eubacteriales; Eubacteriaceae; Rhabdanaerobium</td><td>UA|SA|UAn|SAn</td></tr>
<tr><td>Bacteria; Firmicutes; Bacilli; Bacillales; Planococcaceae; Solibacillus</td><td>UA|SA|UAn|SAn</td></tr>
<tr><td>Bacteria; Firmicutes; Clostridia; Eubacteriales; Peptococcaceae; Pelotomaculum</td><td>UA|SA|UAn|SAn</td></tr>
<tr><td>Bacteria; Firmicutes; Bacilli; Bacillales; Planococcaceae; Sporosarcina</td><td>UA|SA|UAn|SAn</td></tr>
<tr><td>Bacteria; Firmicutes; Clostridia; Eubacteriales; Clostridiaceae; Butyricicoccus</td><td>UA|SA|UAn|SAn</td></tr>
<tr><td>Bacteria; Actinobacteria; Actinomycetia; Kineosporiales; Kineosporiaceae; Angustibacter</td><td>UA|SA|UAn|SAn</td></tr>
<tr><td>Bacteria; Bacteroidetes; Bacteroidia; Bacteroidales; Tannerellaceae; Parabacteroides</td><td>UA|SA|UAn|SAn</td></tr>
<tr><td>Bacteria; Firmicutes; Bacilli; Bacillales; Paenibacillaceae; Cohnella</td><td>UA|SA|UAn|SAn</td></tr>
<tr><td>Bacteria; Firmicutes; Clostridia; Eubacteriales; Oscillospiraceae; Sporobacter</td><td>UA|SA|UAn|SAn</td></tr>
<tr><td>Bacteria; Firmicutes; Bacilli; Bacillales; Bacillaceae; Lottiidibacillus</td><td>UA|SA|UAn|SAn</td></tr>
<tr><td>Bacteria; Firmicutes; Bacilli; Bacillales; Bacillaceae; Priestia</td><td>UA|SA|UAn|SAn</td></tr>
<tr><td>Bacteria; Firmicutes; Clostridia; Eubacteriales; Peptococcaceae; Desulfofundulus</td><td>UA|SA|UAn|SAn</td></tr>
<tr><td>Bacteria; Firmicutes; Clostridia; Eubacteriales; Syntrophomonadaceae; Syntrophomonas</td><td>UA|SA|UAn|SAn</td></tr>
<tr><td>Bacteria; Firmicutes; Bacilli; Bacillales; Paenibacillaceae; Brevibacillus</td><td>UA|SA|UAn|SAn</td></tr>
<tr><td>Bacteria; Firmicutes; Bacilli; Bacillales; Bacillaceae; Massilibacterium</td><td>UA|SA|UAn|SAn</td></tr>
<tr><td>Bacteria; Firmicutes; Bacilli; Lactobacillales; Streptococcaceae; Streptococcus</td><td>UA|SA|UAn|SAn</td></tr>
<tr><td>Bacteria; Firmicutes; Clostridia; Halanaerobiales; Halanaerobiaceae; Halocella</td><td>UA|SA|UAn|SAn</td></tr>
<tr><td>Bacteria; Actinobacteria; Actinomycetia; Jiangellales; Jiangellaceae; Jiangella</td><td>UA|SA|UAn|SAn</td></tr>
<tr><td>Bacteria; Firmicutes; Bacilli; Lactobacillales; Lactobacillaceae; Lactobacillus</td><td>UA|SA|UAn|SAn</td></tr>
<tr><td>Bacteria; Firmicutes; Clostridia; Eubacteriales; Peptococcaceae; Desulfitispora</td><td>UA|SA|UAn|SAn</td></tr>
<tr><td>Bacteria; Firmicutes; Bacilli; Bacillales; Bacillaceae; Mesobacillus</td><td>UA|SA|UAn|SAn</td></tr>
<tr><td>Bacteria; Bacteroidetes; Bacteroidia; Bacteroidales; Bacteroidaceae; Bacteroides</td><td>UA|SA|UAn|SAn</td></tr>
<tr><td>Bacteria; Actinobacteria; Actinomycetia; Streptomycetales; Streptomycetaceae; Streptomyces</td><td>UA|SA|UAn|SAn</td></tr>
<tr><td>Bacteria; Proteobacteria; Deltaproteobacteria; Myxococcales; Phaselicystidaceae; Phaselicystis</td><td>UA|SA|UAn|SAn</td></tr>
<tr><td>Bacteria; Firmicutes; Clostridia; Eubacteriales; Peptococcaceae; Desulfonispora</td><td>UA|SA|UAn|SAn</td></tr>
<tr><td>Bacteria; Actinobacteria; Actinomycetia; Pseudonocardiales; Pseudonocardiaceae; Pseudonocardia</td><td>UA|SA|UAn|SAn</td></tr>
<tr><td>Bacteria; Firmicutes; Bacilli; Bacillales; Bacillaceae; Lentibacillus</td><td>UA|SA|UAn|SAn</td></tr>
<tr><td>Bacteria; Firmicutes; Bacilli; Bacillales; Thermoactinomycetaceae; Thermoflavimicrobium</td><td>UA|SA|UAn|SAn</td></tr>
<tr><td>Bacteria; Firmicutes; Clostridia; Eubacteriales; Clostridiaceae; Caloranaerobacter</td><td>UA|SA|UAn|SAn</td></tr>
<tr><td>Bacteria; Firmicutes; Bacilli; Bacillales; Bacillaceae; Lysinibacillus</td><td>UA|SA|UAn|SAn</td></tr>
<tr><td>Bacteria; Firmicutes; Bacilli; Bacillales; Bacillaceae; Cytobacillus</td><td>UA|SA|UAn|SAn</td></tr>
<tr><td>Bacteria; Firmicutes; Clostridia; Eubacteriales; Clostridiaceae; Hathewaya</td><td>UA|SA|UAn|SAn</td></tr>
<tr><td>Bacteria; Firmicutes; Negativicutes; Veillonellales; Veillonellaceae; Megasphaera</td><td>UA|SA|UAn|SAn</td></tr>
<tr><td>Bacteria; Firmicutes; Bacilli; Lactobacillales; Lactobacillaceae; Limosilactobacillus</td><td>UA|SA|UAn|SAn</td></tr>
<tr><td>Bacteria; Firmicutes; Clostridia; Eubacteriales; Oscillospiraceae; Acetivibrio</td><td>UA|SA|UAn|SAn</td></tr>
<tr><td>Bacteria; Firmicutes; Bacilli; Bacillales; Thermoactinomycetaceae; Kroppenstedtia</td><td>UA|SA|UAn|SAn</td></tr>
<tr><td>Bacteria; Firmicutes; Bacilli; Bacillales; Thermoactinomycetaceae; Melghirimyces</td><td>UA|SA|UAn|SAn</td></tr>
<tr><td>Bacteria; Firmicutes; Clostridia; Eubacteriales; Peptostreptococcaceae; Clostridioides</td><td>UA|SA|UAn|SAn</td></tr>
<tr><td>Bacteria; Proteobacteria; Gammaproteobacteria; Xanthomonadales; Xanthomonadaceae; Luteimonas</td><td>UA|SA|UAn|SAn</td></tr>
<tr><td>Bacteria; Firmicutes; Clostridia; Eubacteriales; Lachnospiraceae; Anaerocolumna</td><td>UA|SA|UAn|SAn</td></tr>
<tr><td>Bacteria; Firmicutes; Bacilli; Bacillales; Planococcaceae; Rummeliibacillus</td><td>UA|SA|UAn|SAn</td></tr>
<tr><td>Bacteria; Firmicutes; Clostridia; Eubacteriales; Peptostreptococcaceae; Intestinibacter</td><td>UA|SA|UAn|SAn</td></tr>
<tr><td>Bacteria; Firmicutes; Clostridia; Eubacteriales; Unclassified Eubacteriales; Flintibacter</td><td>UA|SA|UAn|SAn</td></tr>
<tr><td>Bacteria; Acidobacteria; Vicinamibacteria; Unclassified Vicinamibacteria; Vicinamibacteraceae; Vicinamibacter</td><td>UA|SA|UAn|SAn</td></tr>
<tr><td>Bacteria; Firmicutes; Clostridia; Eubacteriales; Peptococcaceae; Desulfohalotomaculum</td><td>UA|SA|UAn|SAn</td></tr>
<tr><td>Bacteria; Firmicutes; Bacilli; Bacillales; Alicyclobacillaceae; Alicyclobacillus</td><td>UA|SA|UAn|SAn</td></tr>
<tr><td>Bacteria; Firmicutes; Clostridia; Eubacteriales; Clostridiaceae; Alkaliphilus</td><td>UA|SA|UAn|SAn</td></tr>
<tr><td>Bacteria; Chloroflexi; Thermomicrobia; Sphaerobacterales; Sphaerobacteraceae; Sphaerobacter</td><td>UA|SA|UAn|SAn</td></tr>
</table>
